# Self-Healing of Polymers and Polymer Composites

**DOI:** 10.3390/polym14245404

**Published:** 2022-12-09

**Authors:** Vadim I. Irzhak, Igor E. Uflyand, Gulzhian I. Dzhardimalieva

**Affiliations:** 1 Federal Research Center of Problems of Chemical Physics and Medicinal Chemistry, Russian Academy of Sciences, 142432 Chernogolovka, Russia; 2 Department of Chemistry, Southern Federal University, 344090 Rostov-on-Don, Russia; 3 Moscow Aviation Institute, National Research University, 125993 Moscow, Russia

**Keywords:** self-healing materials, self-healing polymers, covalent adaptable networks, vitrimers, repairing capsular components, microvascular networks, non-covalent healing, self-healing metallopolymers, metal–ligand coordination interactions

## Abstract

This review is devoted to the description of methods for the self-healing of polymers, polymer composites, and coatings. The self-healing of damages that occur during the operation of the corresponding structures makes it possible to extend the service life of the latter, and in this case, the problem of saving non-renewable resources is simultaneously solved. Two strategies are considered: (a) creating reversible crosslinks in the thermoplastic and (b) introducing a healing agent into cracks. Bond exchange reactions in network polymers (a) proceed as a dissociative process, in which crosslinks are split into their constituent reactive fragments with subsequent regeneration, or as an associative process, the limiting stage of which is the interaction of the reactive end group and the crosslink. The latter process is implemented in vitrimers. Strategy (b) is associated with the use of containers (hollow glass fibers, capsules, microvessels) that burst under the action of a crack. Particular attention is paid to self-healing processes in metallopolymer systems.

## 1. Introduction

The avalanche-like growth in the production and consumption of synthetic polymeric materials, inherent in recent decades, has forced humankind look for ways to dispose of these in case of damage. In particular, the use of thermosetting epoxy resins results in a large amount of waste. Disposal methods, such as landfills and incineration, lead to serious environmental pollution and the waste of resources. In order not to multiply the pollution of the planet, polymers should be repaired or degraded. The first path leads to the development of self-healing methods [1,2,3,4,5,6], and at the same time, the problem of saving non-renewable resources is solved; the second path leads to the creation of materials that are biodegradable or destroyed by light, moisture, and similar climatic factors. According to S. van der Zwaag [7], for twenty centuries, the development of materials has been reduced to the prevention of damage, that is, materials must be designed and manufactured in such a way as to delay the formation and propagation of damage that occurs during the operation of products as much as possible. Currently, “an alternative approach to 20 centuries of materials science” is gaining momentum, namely, the development of self-healing polymers and polymer composites that can heal in response to damage, regardless of their localization in the material and the time of occurrence. The phenomenon of healing is defined as the restoration of the original mechanical properties of the damaged material.

The idea of extending the service life of composite materials through self-healing is largely borrowed from biological systems in which self-healing is an ordinary event (for example, the healing of human skin or tree bark) [8,9,10,11]. As an implementation of this idea, composites with microvascular networks have been developed [12,13,14]. Attention is drawn to self-healing coatings, an integral property of which is the ability to heal damage [13,14,15,16,17]. In particular, such coatings were developed to ensure the corrosion resistance of metal products [15,18,19,20,21].

**Figure 1:**
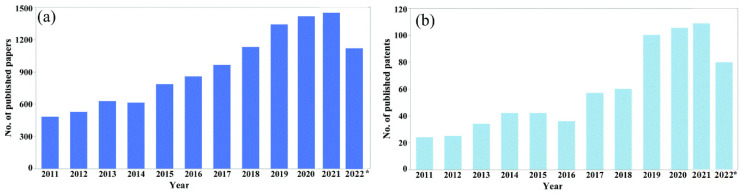
Number of published (**a**) papers and (**b**) patents from 2011 to August 2022 (according to Google Scholar, searched by “self-healing” in the title of the article).

Interest in the problem of self-healing polymers and polymeric materials is continuously growing, as evidenced by the regular increase in the number of publications in the world scientific and patent literature over time (Figure 1).

The purpose of this article is to review the results published in recent years on the self-healing of polymers, polymer composites, and coatings.

## 2. Methods of Self-Healing of Polymeric Materials

Thermoplastic polymers are able to heal when the surfaces of cracks connect, and the polymer chains are entangled again [22]. One of the simple ways to heal cracked or scratched coatings is to apply solvents or heat to the damaged area, which allows for the surface to be wetted and healed, as well as moving and entangling the polymer chains [23]. The healing of weakly crosslinked meshes also occurs at a temperature 10–20° above their glass transition temperature (T_g_). This was demonstrated in the work of [24], where epoxy coatings were prepared by curing diglycidyl ether of bisphenol A (DGEBA) with a mixture of diamine and monoamine in the presence of 0.05–1 phr of carbon black as a photothermal filler. It was shown that an increase in the content of the latter or in the intensity of IR irradiation reduced the healing time.

This approach is unsuitable for thermoplastics and complex multicomponent composites. An analysis of the functional and structural properties of self-healing network polymers [25,26,27] led to the conclusion that their development is reduced to two strategies [8,11,28]: (a) using the reversibility of crosslinks [10,29,30] and (b) introducing a healing agent into cracks [12,31,32,33]. In the latter case, a specific method of vascular self-healing is distinguished [34,35,36,37,38]. In addition, polymer spacers are used in composites [39,40,41].

The first (intrinsic) approach assumes that self-healing occurs due to the chemical bonds of the polymer material itself, which can heal the structure after damage under the influence of external factors such as heat, ultraviolet light, or chemicals. In other words, self-healing networks heal due to their inherent (intrinsic) properties, namely, crosslinks can migrate along the polymer chains between different positions without the risk of structural damage or the loss of material properties.

**Figure 2:**
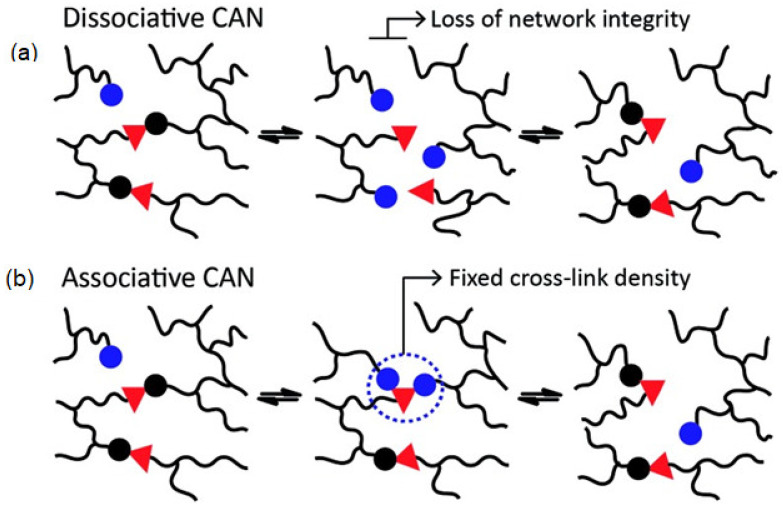
Dissociative (**a**) and associative (**b**) CANs based on exchange reactions and occurring, respectively, with or without a temporary loss of crosslink density. Reproduced from [43]. with permission from the Royal Society of Chemistry.

Polymers containing such dynamic bonds are known as covalent adaptable networks (CANs). Bond exchange reactions in network polymers proceed according to one of two mechanisms [42,43]: a dissociative process, in which crosslinks are split into their constituent reactive fragments with subsequent regeneration [44]; or an associative process, in which a pendant reactive group enters into substitution reactions with an existing crosslink. These mechanisms are realized as interchain exchange reactions [45], in particular, transesterification [46,47] and metathesis [48,49,50]. In any case, the topological structure in CAN is restructured, which is triggered by pressure and temperature and leads to the healing of damages (Figure 2).

Associative CANs belong to the family of dynamic polymers [51] called dynamers [52,53], a term introduced by J.-M. Lehn for polymers, which also includes supramolecular polymers. The dynamic properties of the latter are achieved through intermolecular interactions such as hydrogen bonds, metal–ligand (M–L) coordination, and π–π interactions [39,41,54,55]. The exchange of non-covalent bonds is also used as a dissociative method for solving problems in the self-healing of polymeric materials [51,56].

The second (extrinsic) method consists of the release of healing agents from containers (hollow glass fibers, capsules, microvessels), which break under the action of a crack propagating inside the material and lead to its healing [32,33]. Most of the healing action takes place at room temperature, but sometimes heating is required to improve healing efficiency. Efficiency is measured quantitatively as the ratio of any property of the healed material to the original, expressed as a percentage.

As a rule, the coating is more difficult to heal with the intrinsic method than its bulk counterpart. This fact is explained by the fact that the small thickness of the coating and strong adhesive bond with the substrate limit the mobility of polymer networks, which is necessary to eliminate the damage [37]. Basically, polymer coatings are considered [37,56,57] as autonomous systems that use the external healing method capable of restoring their bulk integrity or functional properties without any external physical intervention. However, the use of containers in thin layers of coatings is limited by the size of the inclusions. Thus, in the work of [20], nanocapsules with a diameter of 100–800 nm were used.

Some systems use nanoparticles (NPs) that spontaneously migrate into cracks formed because of polymer damage [22,58,59,60]. If CdS NPs are small (3 nm) compared to the radius of gyration (R_g_) of the polymer, the constraints on the chain configuration are small, and therefore the entropy cost (Δ*S*) for incorporating NPs into the polymer matrix is low. However, with larger (5.2 nm) particles (comparable to R_g_), Δ*S* increases, due to which NPs will be more easily pushed out of the matrix into an open crack [61].

Self-healing materials can be repaired by intrinsic methods only after crack closure, that is, their recovery consists of two stages: crack closure and healing. Therefore, the mechanisms of closure and the chemical process of the restoration of polymer structures should be especially considered [62]. It should be noted that self-healing is different from self-adhesion. In the first case, the contacting surfaces are not in equilibrium with respect to the reactive groups, and the second case, the process is in equilibrium. Extrinsic methods require considering the relationship between the choice of the location of functional containers and the localization of stress in the composite [63].

The introduction of thermoplastic polymers, such as copolymers of ethylene with methyl acrylate or methacrylic acid, increases the interlayer fracture toughness of the composites, although it causes a decrease in the interlayer shear strength. Thus, to create a three-dimensional self-healing fiber system that also provides high fracture toughness, filaments from ethylene-methacrylic acid copolymer were sewn into an epoxy carbon fiber laminate [64]. The method of healing with thermoplastics requires the application of heat [65].

Note that despite the development of self-healing methods, it would not hurt to have indicators in polymers and composite materials that can detect mechanical damage and the need for healing before the damage becomes catastrophic. This diagnostic is especially important for the method of intrinsic self-healing, in order to determine the moment at which it is necessary to stimulate the process with pressure and temperature. Research [66] has offered an efficient way to measure the occurrence and propagation of damage by evaluating electrical resistance using embedded networks of carbon nanotubes. The applied deformation leads to an increase in the resistance in these materials due to the piezoresistance of individual NPs and an increase in the tunneling distance between the particles. Damage in the form of matrix cracking breaks electrical contacts, leading to an even more pronounced effect on the electrical resistance value.

## 3. Intrinsic Self-Healing

As mentioned above, to repair damage in the form of a crack by the method of intrinsic self-healing, the convergence of its edges is first required, as is, second, the elimination of the resulting interfaces. The first stage proceeds at an elevated temperature and pressure, and the second stage proceeds at through exchange reactions. Next, the second stage is considered.

### 3.1. Dissociative Approach

#### 3.1.1. Covalent Bonds

When using dissociative CANs, reversible covalent bonds are used, which, under the influence of factors such as light or temperature, come to a dissociation–association equilibrium state (reversible additive rearrangements [67]). Due to the breaking of the crosslinks, the network segments bearing the terminal reactive groups are free to diffuse until they meet and react with each other, regenerating a new crosslink. This repetitive process of dissociation, diffusion, and association occurring throughout the material determines the nature of the deformation of the crosslinked materials.

**Figure 3:**
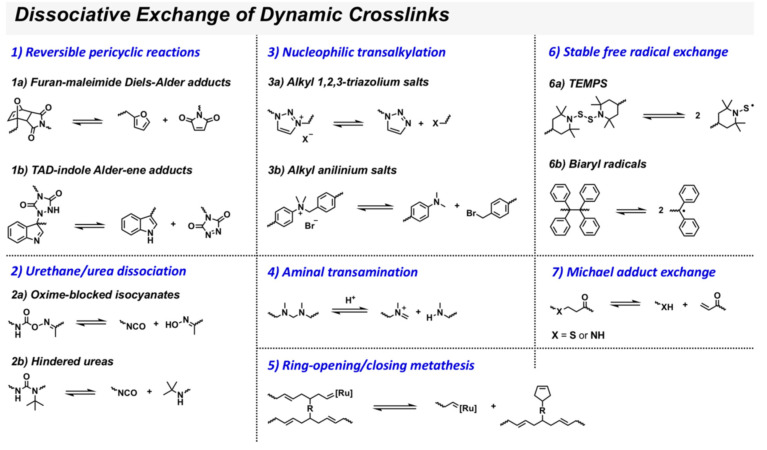
Review of crosslink chemistry used for dissociative covalent adaptive networks (TAD, triazolinedione; TEMPS, tetramethylpiperidine-1-sulfanyl). Reprinted with permission from [68]. Copyright (2019) American Chemical Society.

The effect of temperature is the simplest and most versatile way to modify chemical processes; therefore, thermoreversible networks are the most common type of dissociative covalent adaptable materials [68]. Examples of bond-breaking equilibria are shown in Figure 3. The incorporation of such reactive groups into thermoset polymers (e.g., a disulfide bond) gives them the ability to heal damage. Since the crosslink density of thermoreversible dissociative CANs is temperature-dependent due to thermodynamic equilibrium (see Figure 3), their mechanical and rheological properties are also controlled by temperature.

Due to this dynamic behavior, the crosslinked polymers exhibit not only shape adaptability and remoldability at temperatures above T_g_, but also solid-state plasticity. Although temperature is a universal stimulus to drive or control dynamic exchange processes in most CANs, it is important that any thermal treatment method be effective for several cycles of self-healing without polymer degradation, change in mechanical properties, or loss of appearance of the product. P. Fan et al. [14] developed a recoverable rigid polymer based on dynamic Si-OR and Si-O-Si covalent bonds that are formed with water at room temperature at the site of sample damage. Above T_g_, these bonds and sufficient chain mobility lead to self-healing, the efficiency of which in terms of strength was 72.8% at 25 °C and 99.6% at 70 °C.

Piperazine-based elastomeric polyurethanes synthesized in the work of [69] have excellent mechanical properties and exceptional processability due to the presence of dynamic type 2b urea bonds (see Figure 3).

Photoinduced processes based on the photoreversible breaking of a covalent bond lead to multiple reactions for each absorbed photon. Allyl sulfides and disulfides are typical reagents that dissociate under such an influence. The latter are most popular as dissociative polymeric materials [70]. This is due to the relative weakness of type 6a S-S bonds (see Figure 3), which are reversibly broken under the action of light, after which the radicals either recombine to form a new disulfide (dissociative mechanism) or enter a chain transfer reaction with other disulfides (associative mechanism). The presence of carbon radicals, nucleophiles, or free thiols affects the kinetics of the disulfide rearrangement, changing the mechanism from dissociative to associative [67]. Polymethacrylates containing thermally exchangeable bis(2,2,6,6-tetramethylpiperidin-1-yl)disulfide as a crosslinking site are typical dissociative CANs, easily cured and processed materials [71].

#### 3.1.2. Non-Covalent Interactions

Non-covalent bonding is realized through directional interactions such as hydrophobic effects, hydrogen bonds, M–L coordination, electrostatic, ionic, π–π, and host–guest interactions [23]. Their applicability for self-healing composites is due to their reversibility and speed, directivity and sensitivity, and the ability to respond to weak impacts [25]. Among them, hydrogen bonds are considered the most promising due to their highly dynamic response to external stimuli, combined with a directed and tunable self-association strength [72].

**Scheme 1:**
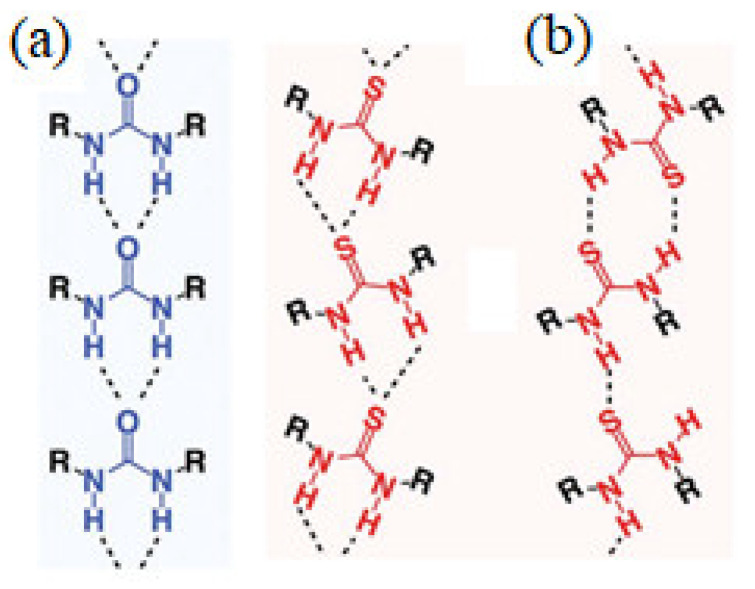
Hydrogen bond structures: (**a**) linear ureas and (**b**) zigzag thioureas. Reprinted with permission from Ref. [73]. Copyright (2022) the American Association for the Advancement of Science (AAAS).

Low-molecular weight (*M_n_* ~1000, *M_w_* ~2000) polyetherthioureas crosslinked by hydrogen bonds, despite extremely slow diffusion dynamics, provide mechanically strong, easily recoverable materials [73]. Due to the zigzag structure of hydrogen bonds in thiourea (Scheme 1b), crystal formation does not occur. A feature of the system was the inclusion of a structural element (triethylene glycol) to activate the exchange of hydrogen bonds, which made it easy to connect the edges of cracks during compression.

T. Liu et al. [72] reported on the development of a self-healing epoxy coating in which 2-ureido-4[1H]-pyrimidinone is grafted to epoxy matrix macromolecules as a quadrupole hydrogen bond using an amino-terminated propylene glycol oligomer. Without any external impact, the epoxy matrix self-heals for ~5 min with an efficiency of more than 70%.

Molecules that form both chains and crosslinks due to hydrogen bonds were synthesized [74]. Three types of functional groups were obtained by a two-step synthesis, namely amidoethylimidazolidone, di(amidoethyl)-urea, and diamidotetraethyltriurea. In the first step, the acidic groups were condensed with a controlled excess of diethylenetriamine. In the second step, the resulting product reacted with urea. Translucent glassy plastic, when heated to 90 °C, significantly exceeding its glass transition temperature (T_g_ = 28 °C), behaves like soft rubber with a deformation at a break of about 350% and completely restores its dimensions after deformation up to 100%.

D.W.R. Balkenende et al. [75] mixed high- and low-molecular weight copolymers of DGEBA with isophthalic-terminated epichlorohydrin in different ratios. The mixture was combined with pyridine, maintaining a 1:1 molar ratio between -COOH and pyridine groups. As a result, supramolecular reticulated glasses with high T_g_ (73–100 °C) were obtained by assembling multifunctional blocks with a high weight fraction of reversible isophthalic acid-pyridine bonds. The shear strength and modulus of the original and processed samples were ~1.4 MPa and ~3.7 GPa, respectively.

Complexes of cyclodextrins (CDs) with non-covalent host–guest interactions are attracting attention due to their biocompatibility, ease of modification, accessibility, reversible nature, and the possibility of complexation with various guest molecules. In the work of [76], to impart self-healing properties to the composite, host–guest chemistry was used to include the corresponding non-covalent bond in the epoxyacrylate structure. The “host” was 6-glycidylmethacrylate-β-CD, and the “guest” was acrylamidoazobenzene. The complex of these compounds served as a crosslinking agent for the copolymer of butyl acrylate with epoxyacrylate.

The article in [77] is devoted to a review of various guest molecules. Each guest molecule imparts unique properties to supramolecular self-healing materials. For example, adamantane, ferrocene, azobenzene, and cholic acid cause high stability, electrochemical sensitivity, photosensitivity, and biocompatibility. It was shown in the work of [78] that epoxy composites with β-CD-graphene crosslink have a high healing efficiency of up to 79.2% and a tensile strength of up to 20.8 MPa upon heating or IR irradiation.

**Scheme 2:**
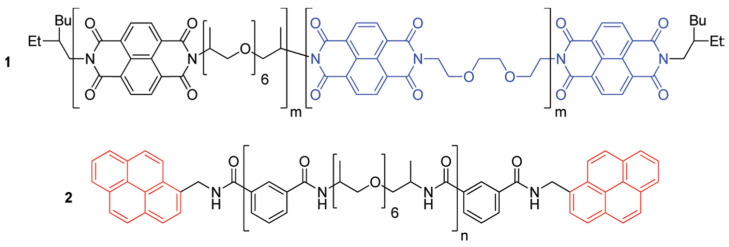
Molecular structure of a 1 + 2 self-healing polymer blend. Reprinted with permission from [55]. Copyright (2012) American Chemical Society.

The supramolecular polymer mixture formed by the π–π interaction between a pyrenyl oligomer (1) with end blocks rich in π-electrons and an oligomer containing naphthalene-diimide units (2) poor in π-electrons was reinforced with cellulose nanocrystals to obtain a self-healing nanocomposite (Scheme 2) [55].

Epoxy polymers with non-covalent interactions are usually brittle and break under heavy loads [24].

### 3.2. Associative Approach: Vitrimers

Associative CANs, known as vitrimers [79,80,81,82], in contrast to dissociative networks, are characterized by the possibility of exchange substitution reactions, which ensures that the degree of crosslinking is independent of temperature [82]. They are often considered [43,83,84] as special materials that combine the properties of a thermoplastic and a thermoset with a topological transition temperature (*T_v_)*: at *T* ≥ *T_v_* > *T_g_,* an exchange of bonds occurs, leading to a flow to the polymer, and the material passes from a viscoelastic solid (thermoset) into a viscoelastic fluid (thermoplastic). In vitrimers, bond exchange occurs within a single reaction [29,67,68,85]. The most common type of dynamic covalent exchange in esters and related compounds, such as carbamates, carbonates and carbamides, as well as acetals and imines, is “addition-elimination” [84].

An essential feature of polymer systems in which an interchain exchange reaction occurs is that each dead polymer chain can become alive upon interaction with the corresponding agent [45]. This approach was first used by L. Leibler et al. [79] to obtain network polymers that are glassy at room temperature, but with the possibility of rearranging the topological structure into a highly elastic state, which is why they are called vitrimers [80]. At the same time, the proportion of crosslinks, the nature of which allows an exchange reaction, does not have to be 100%: since the proportion of crosslinks that cannot be exchanged is insufficient to reach the gelation point, the vitrimer can be processed with complete restoration of the crosslink density [62,86]. Thus, at a critical fraction of 50 mol.% of a vitrimer containing 40 mol.% of permanent crosslinks, after re-treatment, the complete restoration of mechanical properties is achieved, and creep decreases by 65–71% [87]. The increased creep resistance achieved due to the topological structure of the chain of block vitrimers can be used to control the viscoelastic properties [88]. This strategy is applicable for converting non-recyclable, permanently crosslinked thermosets into vitrimer-type dynamic nets with reusability.

In composites at the interface, vitrimers can form new chemical bonds through exchange reactions, whereas in standard thermoset materials, chemical bonding can only occur if there are remaining reactive groups due to an incomplete cure [89].

#### 3.2.1. Vitrimers Based on the Transesterification Reaction

**Figure 4:**
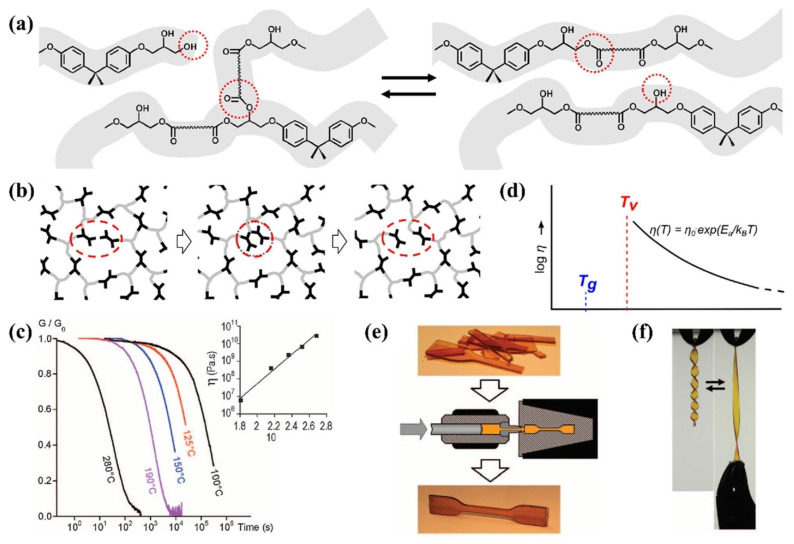
(**a**) An illustration of transesterification in hydroxyl ether networks. (**b**) Topological rearrangements through exchange reactions that preserve the integrity of the network. (**c**) Normalized stress relaxation at various temperatures. The inset shows the change in viscosity with temperature at zero shear. (**d**) The viscosity of the vitrimer follows the Arrhenius law with increasing temperature. (**e**) Remachining broken parts on an injection molding machine to restore their original appearance and properties. (**f**) Shaping the sample into a fusilli shape. Reprinted with permission from [79]. Copyright (2022) the American Association for the Advancement of Science (AAAS) and from [68]. Copyright (2019) American Chemical Society.

In a pioneering work [79], a crosslinked polymer was obtained by the reaction of DGEBA and a mixture of dicarboxylic and tricarboxylic acids with a 1:1 epoxy group/COOH stoichiometric ratio. The presence of hydroxyl and ester groups during catalysis with zinc acetylacetonate ensured the transesterification reaction. The synthesized material in the glassy state was identical in properties to the classical epoxy anhydride polymer: T_g_ ≅ 80 °C, Young’s modulus ~1.8 GPa, and the stress at the break was ~55 MPa. However, unlike the latter, at *T* ≫ T_g_, the material exhibits fluidity due to the transesterification reaction and the rearrangement of the network topological structure caused by it (Figure 4).

At 200 °C, a network polymer containing 10 mol.% catalyst deforms linearly with time in a creep experiment after a transient regime. Viscosity estimated from the slope of the straight line is ~1.2 × 10^10^ Pa·s, and measured over a wide temperature range, it shows an Arrhenius dependence with an activation energy of ~88 kJ/mol. We draw attention to the fact that the viscosity of polymers is usually described by the Williams–Landel–Ferry equation and not by Arrhenius. Consequently, this phenomenon is not related to the free volume of the system but has a purely chemical nature.

In addition to temperature, an important parameter that determines the kinetics of transesterification is the concentration and nature of the catalyst [90,91]. In epoxy vitrimers, zinc acetate is often used to catalyze transesterification due to its low cost, non-toxicity, and high efficiency.

One typical accelerator for the anhydride curing of epoxy resins is 2,4,6-tris(dimethylaminomethyl)phenol (DMP-30), which also acts as a transesterification catalyst. In this case, it plays a dual role: tertiary amines and hydroxyl groups participate in the curing reaction of DHEBA with glutaric anhydride, and tertiary amines catalyze the exchange reaction between ester bonds and hydroxyl groups [92]. DMP-30 allows polymer healing at temperatures below 200 °C and normal pressure.

**Figure 5:**
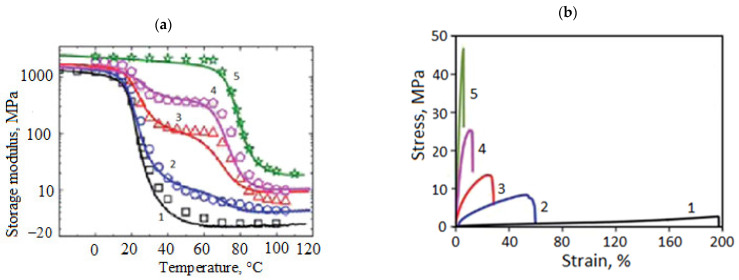
Data on the mechanical properties of the formed vitrimers: (**a**) curves of the temperature dependence of the Young’s modulus (lines show the experimental results, dots show the calculated ones); (**b**) stress-strain curves. (1) A soft component of the vitrimer blend; (2) Soft:Hard = 7:3; (3) Soft:Hard = 5:5; (4) Soft:Hard = 3:71; (5) a hard component of the vitrimer blend. Reproduced from Ref. [93] with permission from the Royal Society of Chemistry.

B. Zhang et al. [93] studied the efficiency of vitrimer healing by synthesizing two polymers of the following composition: DGEBA with glutaric anhydride at an epoxy/acid ratio of 1:1 in the presence of 10 mol.% zinc acetylacetonate (weakly crosslinked, soft) and the system described above [79] (strongly crosslinked, hard). In both polymers, the transesterification reaction proceeded at an elevated temperature. The resulting vitrimers were ground into powder in a rotary mill and mixed in various weight ratios, followed by hot pressing at constant pressure. Figure 5 shows the experimental and calculated data on the mechanical properties of the resulting vitrimers. The authors used finite element modeling of material parameters. The calculation model considered the thermomechanical behavior of pure vitrimers and the random distribution of particles in mixtures. In addition, it was assumed that neighboring particles are well-interconnected, so the effect of interface defects can be neglected. The achieved agreement between experiment and calculation (Figure 5a) confirms the validity of the assumptions included in the model, in particular, the presence of efficient welding of vitrimer particles. As shown in Figure 5b, the blend composition has a strong influence on the stiffness and deformability of vitrimers. The more the soft component in the blend, the lower the modulus of elasticity and the greater the tensile strain.

The topological transition temperature (T_v_) of the epoxy vitrimer, measured by dilatometry, decreases with increasing catalyst concentration. In a vitrimer, a higher coefficient of thermal expansion is observed compared to a permanently crosslinked network [90,94]. The concentration of hydroxyl groups plays a key role. Thus, the inclusion of cellulose nanocrystals as a carrier of hydroxyl groups into the system increases the rate of the transesterification reaction [91,95].

Epoxy coatings were developed [96] containing halloysite nanotubes functionalized with silane and cellulose and exhibiting vitrimer-like behavior due to the transesterification reaction of β-hydroxyether bonds, with the functional groups on the surface of NPs playing a key role as crosslinking agents.

In the work of [97], epoxy nanocomposites with multiwalled carbon nanotubes were obtained, which exhibited the properties of a vitrimer with an associative dynamic rearrangement of bonds upon catalysis by zinc acetate dihydrate. Mechanical strength swa achieved through decomposition and repolymerization of the β-hydroxyl ether at the interface. The samples were successfully processed by repeated pressing of crushed composite particles at 180 °C under a pressure of 4 MPa for 15 min.

The curing of the diepoxide of the tetrahydrobenzyl ester of tetrahydrobenzoic acid (I) with hexahydro-4-methylphthalic anhydride (II) in the presence of zinc acetylacetonate also gave vitrimers [98], but their properties, including the T_v_ value, depend on the I:II ratio. Since there were no hydroxyl groups in the monomer structure, the authors explained this by the hydrolysis of the anhydride by atmospheric moisture.

L. Yue et al. [94] proposed a method for the reduction of thermosetting epoxides by mechanochemical vitrimerization. DGEBA, glutaric anhydride, and imidazole were homogeneously mixed and cured to ensure complete crosslinking. The cured epoxy resin was first ground and then ball milled with zinc acetate to obtain vitrimerized fine powders. After drying, they were placed in a stainless-steel mold and pressed at 250 °C. The resulting vitrimerized epoxy resin underwent a transesterification reaction and welding mechanism in the same way as a synthetic epoxy vitrimer. Increasing the processing temperature, time, or pressure generally resulted in maximum properties. It was shown that the degree of crosslinking and the glass transition temperature have a greater effect on the efficiency of vitrimerization than the concentration of HO-groups.

#### 3.2.2. Vitrimers Based on Disulfide Exchange

Epoxy polymers and matrices containing –S–S groups have been studied in a number of works, for example, in [99,100,101,102]. Disulfide exchange underlies the synthesis of so-called vitrimer-like materials. In the work of [100], along with the problems of the dynamic covalent chemistry of disulfide exchange reactions, noncovalent interactions are also discussed.

The mechanism of disulfide exchange was studied by J.M. Matxain et al. [103]. By analyzing the electronic structure of the transition complex and calculating the corresponding energy barrier for the reaction, the authors concluded that the probability of the reaction occurring—

R_1_S-SR_2_ + R_3_S-SR_4_ ↔ R_1_S-SR_3_ + R_4_S-SR_2_

—directly, by metathesis, is extremely small. According to the authors, the most probable most probable route is a three-stage mechanism, the first stage of which is the decay of the S-S bond:

R_1_S-SR_2_ ↔ R_1_S^•^ + ^•^SR_2_

This is followed by the attack of the radical on the S-S bond:

R_1_S^•^ + R_3_S-SR_4_ ↔ R_1_S-SR_3_ + ^•^SR_4_,

and finally, the recombination of radicals:

R_4_S^•^ + ^•^SR_2_ ↔ R_4_S-SR_2_

Easily processed composites based on degradable thermosetting binders should contain various controlled cleavable bonds, such as, for example, disulfide bonds. The problem is that the plastic must be stable under operating conditions, that is, the bonds introduced must not break up uncontrollably. Thus, the exchange reaction between two disulfide-containing hardeners of epoxidized vegetable oil, 2,2′-dithiodibenzoic and 3,3′-dithiodibenzoic acid, in the presence of imidazole proceeds at 170 °C, and not at 130 °C, i.e., the curing temperature [104].

The inclusion of aromatic disulfide bridges into polymer networks has led to the development of self-healing materials [101]. This allows the cured resin to be used as a raw material for the manufacture of composites. Unlike most cured elastomeric materials, this material can be recovered and recycled in industrial processes, helping to achieve zero waste targets. So, for example, as a result of mixing two different vitrimer networks at 150 °C, a homogeneous material was obtained. The processability of the dynamic epoxy network was studied in hot pressing experiments at 200 °C and 100 bar for 5 min. In this case, the reference material crumbled into powder, and the vitrimer was deformed without being destroyed [101].

The introduction of tetrakis-(3-mercaptopropionate) pentaerythritol into the matrix, that is, the inclusion of disulfide groups in the network chains, led to the renewal of crosslinks on damaged surfaces. Damage was healed at moderate temperatures and led to a complete restoration of mechanical properties [99].

**Figure 6:**
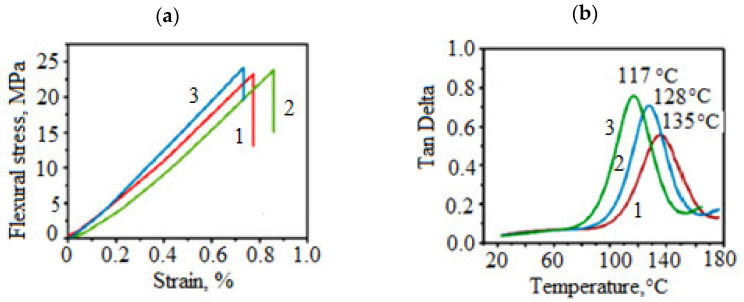
(**a**) Curves of bending stress versus strain of original and remanufactured composites. 1—pristine, 2—1st reprocess, 3—2nd reprocess. (**b**) Tanδ versus temperature curves for the original and processed composites. 1—Pristine, 2—1st reprocess, 3—2nd reprocess. Data of [29].

A typical example is the work of [29], which shows that an epoxy composite with disulfide bonds can be processed into new bulk samples by hot pressing at 150 °C and a pressure of 0.3 MPa for 1 h. This process can be repeated several times (Figure 6). The composite is also amenable to healing. As can be seen from Figure 6, the restored specimens even show a slightly increased bending stress compared to the original specimen.

A. Ruiz de Luzuriaga et al. [102] obtained a fiber-reinforced polymer composite with a degradable epoxy matrix by curing DGEBA with 4-aminophenyl disulfide. Comparison with the composite prepared with diethyltoluenediamine hardener showed their similarity in properties in the glassy state (T_g_ is 130 °C for the first composite and ~127 °C for the second composite). However, after processing in a hot press at 200 °C and 100 bar for 5 min, the second fiber-reinforced polymer composite broke, and the first composite gave a modified object in the form of a film.

In the work of [105], vitrimer (1) was synthesized by the interaction of DGEBA and 4,4′-dithiodibutyric acid at 180 °C in the presence of triazabicyclodecene (TBD) as a transesterification catalyst. The hydroxyl groups formed by the reaction of the epoxy ring, and the acid continued to react with the epoxy resin, resulting in the formation of both crosslinked and branched chains. In the absence of TBD, the synthesis led to the formation of only a crosslinked vitrimer (2). Vitrimer (3) was also obtained by curing DGEBA with sebacic acid. The exchange reactions in the first vitrimer proceed faster than in the second and third, and the rearrangement of polymer chains that occurs in this case is more efficient, which indicates the synergism of the reactions of disulfide exchange and carboxylate transesterification. Unlike the other two vitrimers, the first vitrimer could be effectively processed at 100 °C in 1 h, and no significant strength loss was observed after four cycles.

#### 3.2.3. Vitrimers Based on Dioxaborolanes

**Scheme 3:**
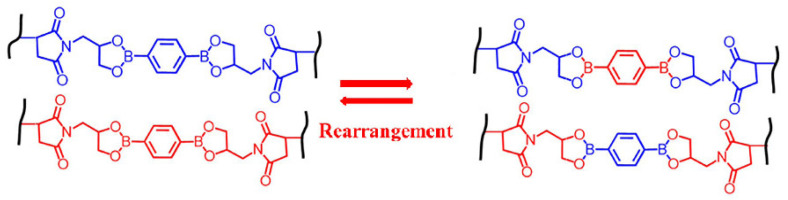
Exchange of dioxaborolanes by metathesis. Reproduced with permission from [106]. Copyright (2022) the American Association for the Advancement of Science (AAAS).

L. Leibler and his collaborators in 2017 proposed a new class of vitrimers based on the use of dioxaborolanes, which made it possible to carry out the bond exchange reaction without a catalyst (Scheme 3) [106].

In a review by M. Hayashi [50], it was shown that the metathesis of dioxaborolanes is the main mechanism of the self-healing of vitrimers obtained from such polymers as polyesters, polylactides, polycarbonates, polydimethylsiloxanes, polydienes, polyurethanes, polyolefins, poly(meth)acrylates, and polystyrenes. They can be recycled many times by extrusion or injection molding without compromising their thermal stability.

In the work of [107], vitrimers were obtained in two stages. The first radical grafting reaction led to the formation of functionalized polymers with lateral dioxaborolane units. At the second stage, the latter reacted with bis-dioxaborolanes in a metathesis reaction, crosslinking polymer chains. However, in a study [108] to obtain vitrimers by the one-stage reactive extrusion of commercial polyethylene, it was possible to combine nitroxyl chemistry for the radical grafting and metathesis of boronic esters as an associative exchange reaction, synthesizing 2,2,6,6-tetramethyl-4-((2-phenyl-1,3,2-dioxaborolan-4-yl)-methoxy)-pi-peridin-1-oxyl.

The kinetics of the exchange reaction in the absence of any catalysts at various temperatures are well-described by a simple second-order model following from the metathesis mechanism. The found activation energy is ~15.9 kJ/mol [106].

**Figure 7:**
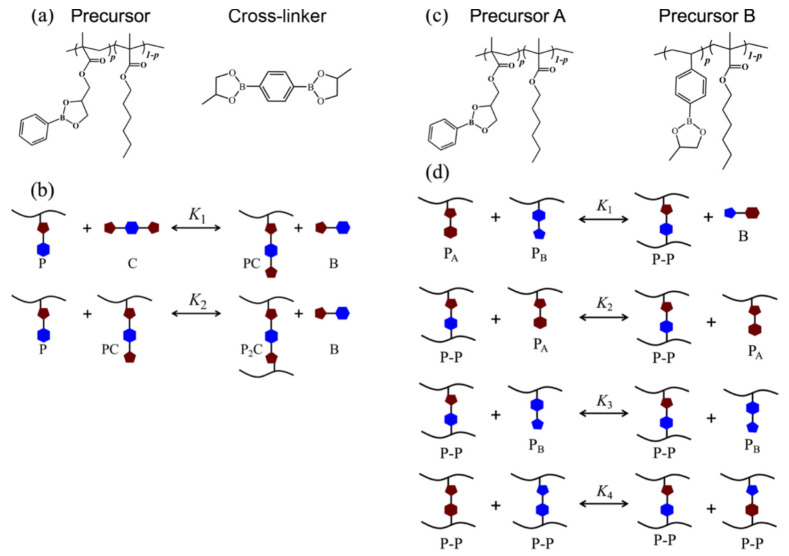
(**a**) Precursor and crosslinker structures and (**b**) two-stage reactions between them [110] and (**c**) structures of precursors A and B and (**d**) dominant reactions between them [109]. Reprinted with permission from [110]. Copyright (2021) American Chemical Society and [109]. Copyright (2020) American Chemical Society.

S. Wu et al. [109,110] studied the thermodynamics and kinetics of the synthesis of vitrimers by the exchange reaction of dioxaborolane metathesis. The processes considered in the works are schematically presented in Figure 7. The model of reversible gelation (Figure 7b) made it possible to estimate the density of crosslinking sites and the equilibrium constants of metathesis reactions of the first and second stages: K_1_ ≈ 1 and K_2_ ≈ 10. The value of the second constant indicates that at this stage, there is an increase in entropy, apparently due to the formation of a free small molecule of the byproduct. In the process of crosslinking, pendant non-crosslinking fragments are formed with an excess of a crosslinking agent, which leads to an additional transition of the gel into a sol. However, at low temperatures, this process is less pronounced, and the degree of crosslinking of the vitrimers is determined by the concentration of the crosslinking agent. This fact was explained in the work of [110] by the temperature dependence of K_2_, and in the work of [107] by the competition between the conformational entropy of polymers and the translational entropy of the crosslinking agent.

A pair of polymeric precursors (Figure 7c) can be considered as one crosslinker in relation to the other (Figure 7d). After crosslinking in the first step, the low-molecular weight byproduct was removed in vacuum at 100 °C for more than one week. Further, the process was determined by the reactions of the second, third, and fourth stages. At a fixed concentration *P_B_*_0_ = 1 M and a change in *P_A_*_0_ from 0 to 4 M, *K*_2_ = *K*_3_ = 1 c^−1^·M^−1^ and *K*_4_ = 0.1, 1 и 10 c^−1^·M^−1^, depending on the value of *P_A_*_0_.

When a boronic acid ester bond is introduced as a crosslink into thermoplastic elastomers with a crystalline phase, such as a copolymer of ethylene with α-olefins, the kinetics of exchange reactions in vitrimers are affected by the crystallinity of the polymer [111]. Due to the restriction of mobility by the crystalline phases, the activation energy E_a_, obtained from the stress relaxation data, is 139 kJ/mol (for a non-crosslinked polymer, E_a_ = 122.2 kJ/mol), and after thermal treatment, which resulted in the elimination of crystallinity, E_a_ = 50.7 kJ/mol.

**Scheme 4:**
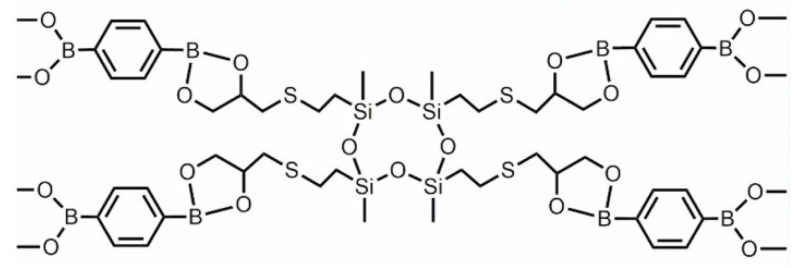
Polydioxaborolane crosslink. Reproduced with permission from [85]. Copyright (2022) Elsevier.

The reactions of the dynamic metathesis of dioxaborolane and the reversible hydrolysis of boronic acid esters make the polydioxaborolane vitrimer with ester bonds of boronic acid in network chains and the possibility of processing under the action of heat or moisture (Scheme 4) [85].

According to a hypothetical estimate, the topological transition temperature (T_v_) of network polydioxaborolane (–16 °C) is lower than its glass transition temperature (~26 °C). Nevertheless, it can be reformed in the temperature range from T_v_ to T_g_ even without the participation of water: under stress, crosslinks (nodes) are able to dissociate, entering exchange reactions.

#### 3.2.4. Vitrimers of Other Structures

**Scheme 5:**
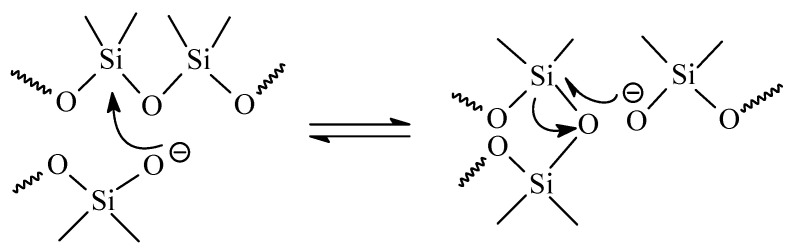
Proposed mechanism for dynamic crosslinking of ionic polydimethylsiloxane networks. Reprinted with permission from [112]. Copyright (2015) American Chemical Society.

The exchange reaction in the polydimethylsiloxane network proceeds at room temperature according to a mechanism such as transesterification (Scheme 5) [112]. The anionic end group breaks the silicon–oxygen bond through a nucleophilic attack, so that a new ≡Si–O- bond and a new anionic end of the chain are formed. The latter can also react elsewhere in the network.

The incorporation of silyl ether bonds into covalently crosslinked polymers leads to vitrimers with dynamic covalent bonds that exhibit both plasticity and processability [113]. Thermosetting epoxides containing siloxane can be prepared over a wide range of temperatures. A mixture of DGEBA and polypropylene glycol diglycidyl ether reacted with the silane coupling agent (3-aminopropyl)-triethoxysilane. A certain amount of KOH was used to obtain dynamic silyl ether bonds [114]. Bis(potassium)oligoaminopropylmethylsiloxane diolate (K-PAMS) was synthesized in the work [115]. The pendent aminopropyl groups, potassium silanolate end groups, and polysiloxane units included in K-PAMS ensured that the latter served both as a DGEBA curing agent and created a dynamic bond through the chemical equilibrium of the siloxane.

**Scheme 6:**
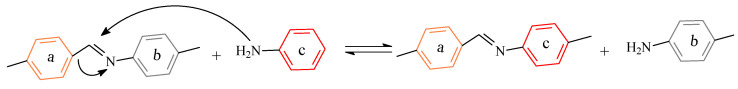
Imine exchange in an epoxy polymer network. Reprinted with permission from [116]. Copyright (2018) American Chemical Society.

Vitrimerization due to imine exchange reactions under the action of residual unreacted primary amino groups (Scheme 6) proceeds at an elevated temperature. The thermosetting polymer was obtained based on vanillin-containing epoxy bisphenol [116].

**Figure 8:**
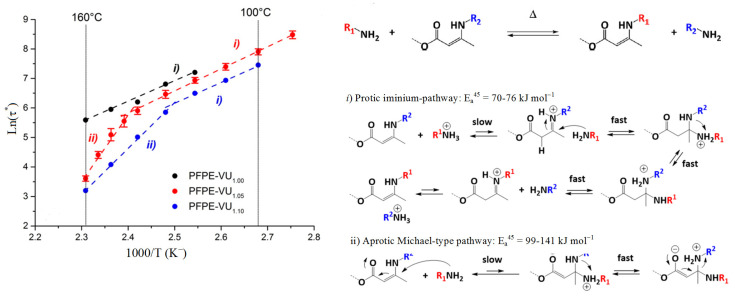
Vinyl urethane vitrimers exhibiting a dual temperature reaction based on two concomitant exchange mechanisms: the iminium type in the lower temperature range with an energy barrier of E_a_ 60–70 kJ/mol and the Michael reaction type at higher temperatures with a higher E_a_ of 130–170 kJ/mol. Reprinted with permission from [117]. Copyright (2018) American Chemical Society.

By crosslinking bisperfluoropolyetheracetoacetate with tris(2-aminoethyl)amine, a vitrimer is formed [117], in which the exchange of bonds is realized as two competing mechanisms, protic iminium and aprotic Michael-type (Figure 8) [118]. Each of these shows a different temperature dependence associated with a significant difference in activation energy (60–70 versus 130–170 kJ/mol). The low barrier process dominates at low temperatures but is replaced by a high barrier process at higher temperatures.

**Scheme 7:**
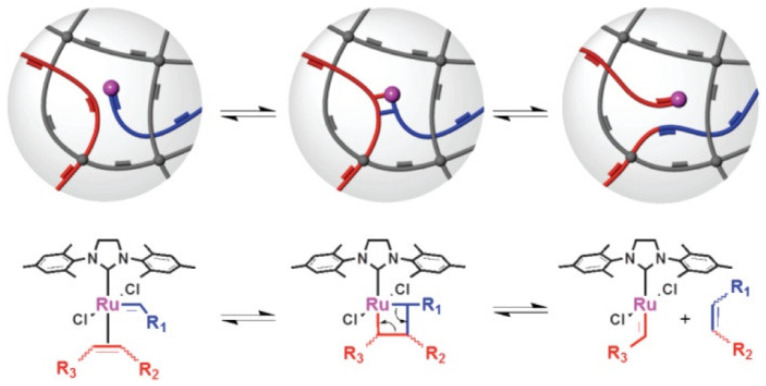
Olefin metathesis. Reprinted with permission from [121]. Copyright (2022) Wiley and Sons.

Z. Guan et al. [119,120] demonstrated the possibility of olefin metathesis vitrimerization in crosslinked polybutadienes containing the Grubbs RuCl_2_(=CHPh)(PCy_3_)_2_ catalyst, which was used due to its high water resistance and good compatibility with hydrocarbons (Scheme 7) [121].

**Scheme 8:**
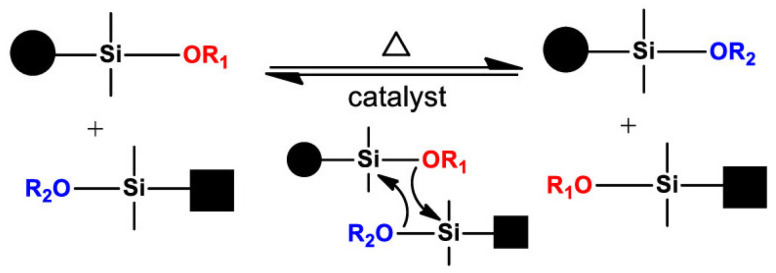
Silyl ether metathesis for vitrimer design. Reprinted with permission from [48]. Copyright (2019) American Chemical Society.

Under anhydrous conditions, a direct exchange of silyl ether groups occurs during catalysis with Bronsted or Lewis acids, demonstrating the reaction of silyl ether metathesis as a dynamic covalent exchange of bonds that ensures plasticity and processability of thermosetting materials (Scheme 8) [48]. In this work, an ethylene-vinyl alcohol copolymer was silylated with trimethylsilyl groups followed by crosslinking with a bis-silyl ether-based reagent. The obtained thermosetting material showed the properties of a vitrimer, that is, plasticity and the ability to be processed at elevated temperatures, but below the melting point, while maintaining high thermal stability.

Vitrimers were obtained in the work in [122] from star-shaped poly((±)-lactide) with terminal hydroxyl groups crosslinked with methylenediphenyl diisocyanate. The materials also showed a short relaxation time, less than 50 s at 140 °C. The vitrimers destroyed by uniaxial tension were healed by pressing and demonstrated a recovery of an ultimate elongation up to 67%, tensile strength up to 102%, and Young’s modulus up to 133%.

**Figure 9:**
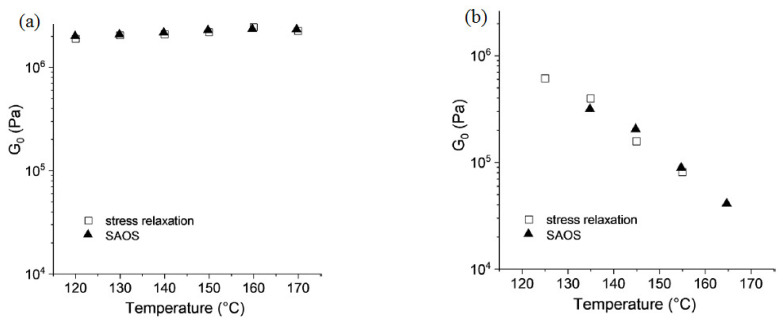
Dependences of the relaxation modulus G_0_ on temperature, obtained by the methods of stress relaxation (1) and low-amplitude vibrational shift (2) for associative I (**a**) and dissociative II (**b**) polymers. Reprinted with permission from [83]. Copyright (2020) American Chemical Society.

Vitrimers, as mentioned above, unlike dissociative CANs, remain completely crosslinked at any temperature. This has been demonstrated by A. Jourdain et al. [83], who compared the relaxation characteristics of a thermoset polymer obtained by the interaction of polystyrene with benzyl iodide side groups with 1,2,3-triazole (I) and a dynamic network based on aliphatic compounds of 1,2,3-triazolium (II). Figure 9 shows data on the dependence of the relaxation modulus G_0_ on temperature obtained by stress relaxation (1) and low-amplitude oscillatory shift (2). As can be seen, in the case of the vitrimer I, the value of G_0_ is constant in a certain temperature range (Figure 9a), whereas the modulus of the dissociative network II decreases with increasing temperature (Figure 9b).

## 4. External Self-Healing

The self-healing strategy used depends on the intended application [63]. For example, to stop slowly propagating cracks, it is enough to correctly distribute the microcapsules, and the embedded hollow glass fibers filled with healing liquid or the vascular network ensure the penetration of the healing agent into large areas of damage.

### 4.1. Use of Microcapsules

Apparently, the very first developments of self-healing materials were based precisely on encapsulated healing agents [123].

**Figure 10:**
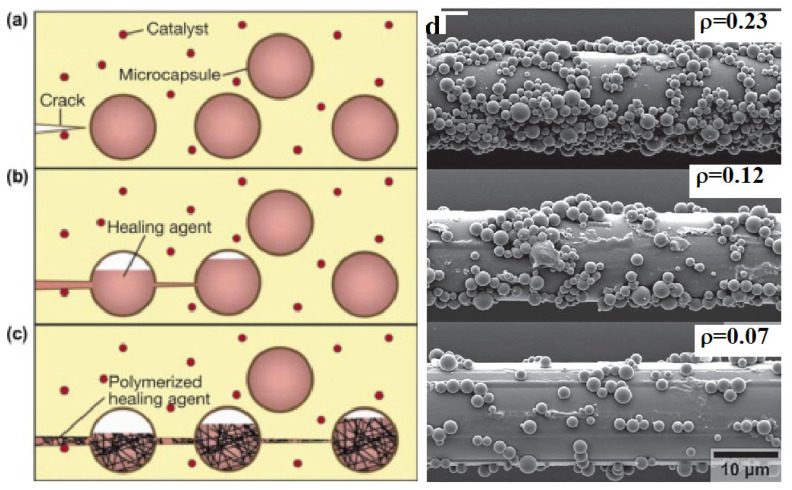
Schematic representation of a self-healing method using microcapsules (**a**–**c**). Reproduced from [127] with permission from Elsevier. SEM images of fibers functionalized with capsules at various concentrations ρ (**d**). Reproduced with permission from [33]. Copyright (2022) John Wiley and Sons.

In the biological world, a self-healing unit is a cell in which various fluids perform certain functions. By analogy, it was proposed to use artificial microcapsules filled with a reagent that can bridge emerging cracks [34]. S.R. White et al. [124] pioneered this approach successfully by embedding encapsulated agents in a polymer matrix containing particulate catalysts, as shown schematically in Figure 10. Dicyclopentadiene (DCPD) and epoxy resin are the most common polymerizable reagents.

The capsules with DCPD and Grubbs’ catalyst (bis(tricyclohexylphosphine) benzylidene ruthenium(IV) dichloride) were dispersed in the polymer matrix during the preparation of the material (Figure 10a). When the material is damaged and cracks appear, the healing agent contained in the capsules is released due to the destruction of the capsule shell and fills the crack under the action of capillary forces (Figure 10b). In this case, the amount of the healing agent delivered to the crack cavity is determined by the probability of the crack meeting with the capsule, which linearly depends on the diameter of the latter; its decrease limits the amount of damage that can be healed. Two different sizes of microcapsules were used to ensure the uniform distribution of microcapsules and adequate delivery of the DCPD healing agent to fracture cavities [125]. When the monomer interacts with the catalyst, the polymerization process proceeds, due to which the crack is healed (Figure 10c). It should be noted that the Grubbs’ catalyst exists in two crystalline polymorphs, each of which differs in crystal shape, thermal stability, and dissolution kinetics. The more rapidly dissolving polymorph demonstrates high healing efficiency [126].

Self-healing model glass-reinforced plastics were obtained [33] by coating fibers with a Grubbs’ catalyst and capsules with a diameter of ~1.5 µm containing the healing monomer DCPD. Figure 10d shows scanning electron microscopy (SEM) images of fiber surfaces with a different capsule surface density (ρ), which is defined as the total number of capsules per 1 µm^2^ of fiber surface area.

To protect the catalyst, it is embedded in wax microspheres [125]. The efficiency of catalysis depends on the size of the sphere: with its increase, due to the decrease in the ratio of surface area to volume, the efficiency decreases.

J. Yamuna et al. [18] studied the process of crack healing in an epoxy coating with poly(aniline formaldehyde) microcapsules encapsulated with linseed oil and mercaptobenzothiazole. To assess the efficiency of self-healing, observation with an optical microscope and the method of a scanning vibrating electrode were used. A coating containing microcapsules can protect steel, since the impedance values remain at the level of 1.29 × 10^8^ ohm·cm^2^ after 15 days of exposure to a 3.5% NaCl solution, and coatings without microcapsules lost their protective ability: already on the third day, the impedance value decreased by ~2 orders of magnitude.

In the work of [128], multimaleimides dissolved in phenylacetate were used as a microencapsulated healing agent; the healing of the thermosetting material occurred due to their reaction with furan dissolved in the matrix. As a result of the introduction of a solution of bismaleimide into the crack surface, covalent Diels–Alder bonds were formed across the crack, and physical adhesion of the crack edges occurred due to their swelling.

S.H. Cho et al. [129] studied the effect on the self-healing efficiency of an epoxy thermoset on the various molecular weights of hydroxyl-end functionalized polydimethylsiloxane, 20% solutions of which in *n*-heptane were encapsulated, as well as the nature of the Sn-based catalyst placed in microcapsules together with the curing agent, the condensation product of toluene-2,4-diisocyanate with 1,4-butanediol. The healing properties of the self-healing composite were improved by reducing the viscosity of the healing agent with an optimal viscosity range of 2000 ÷ 3500 cP (molecular weight 36,000–49,000). The efficiency of healing increased with the use of more active catalysts. Although the reaction mechanism is not well-understood, it has been suggested that the catalytic activity depends primarily on steric and electronic effects.

In the work of [31], the microencapsulation of carbon nanofibers suspended in ethylphenylacetate was used.

**Figure 11:**
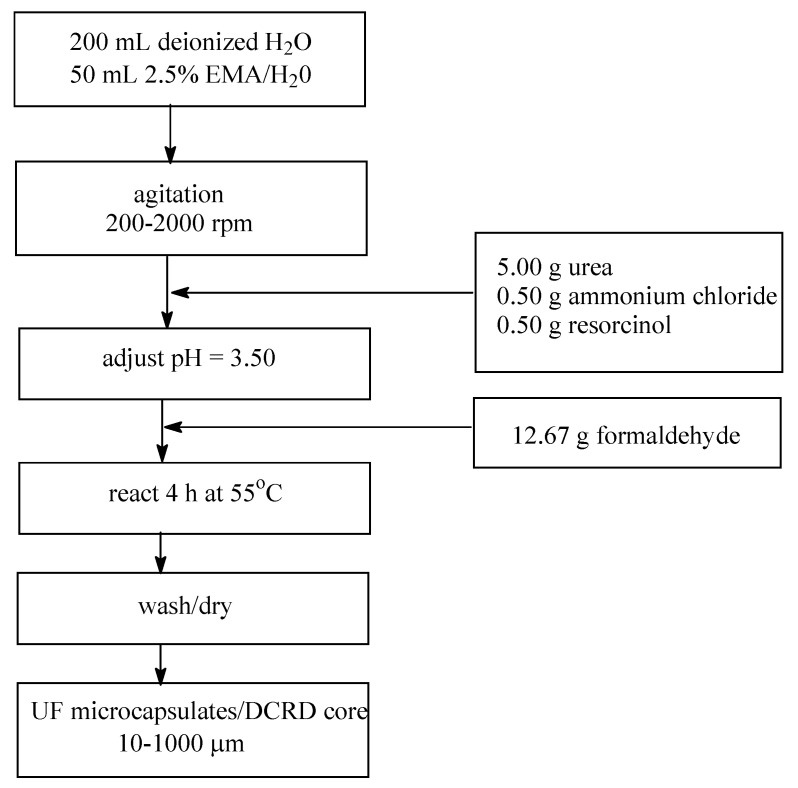
Microencapsulation of DCPD using acid-catalyzed in situ polymerization of urea with formaldehyde to form the capsule wall. EMA is ethylene maleic anhydride. Reproduced from [130] with permission from Taylor & Francis.

Urea-formaldehyde (UF)-coated microcapsules were prepared by in situ polymerization in an oil-in-water emulsion, as shown schematically in Figure 11 [130]. Briefly, the hydrophilic monomers of the shell material are dissolved in water, and a catalyst is added and heated in the presence of all reactants. In the process of polymerization, a solid polymer shell is formed on the surface of a drop of healing material.

Capsule shells play a key role in self-healing processes. They must have stability at elevated temperatures, high interfacial adhesion to the matrix, and the ability to prevent the diffusion of the liquid contained in the microcapsule. The UF shell does not fully satisfy these conditions, but the replacement of urea with melamine [131] in the formulation did not improve the results of self-healing. Therefore, the work in [132] was carried out to create two-layer walls.

The method to produce multilayer capsules includes several separate steps: (1) an interfacial reaction between isocyanates and polyols to create a polyurethane shell wall; (2) washing and filtering the prepared microcapsules; (3) redispersion in an aqueous medium and application of a UF shell. Secondary nucleation on the outside of the microcapsule allows the UF microcapsules to effectively bind to the matrix in the self-healing composite, which is a major problem for single-wall polyurethane capsules.

**Scheme 9:**
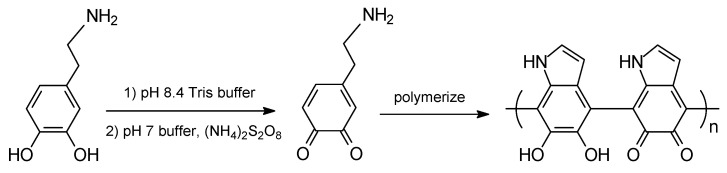
Polymerization of dopamine. Reprinted with permission from [133]. Copyright (2019) American Chemical Society.

S. Kang et al. [133] obtained heat-resistant and solvent-resistant microcapsules with a polymer shell based on dopamine (4-(2-aminoethyl)-1,2-benzene-diol). The schematic diagram is the same as in Figure 11, with a slight difference: instead of the condensation of urea with formaldehyde, the polymerization of dopamine occurs (Scheme 9).

Polymethyl methacrylate (PMMA) and polyetheramine in an oil-in-water emulsion form microcapsules, the first as a shell, the second as a healing agent (epoxy resin hardener) [134]. The thickness of the shell wall, the content of the hardener, and the size distribution of microcapsules were controlled by the conditions of their synthesis. The average diameter increased with increasing temperature and decreased with increasing agitation speed and emulsifier concentration. The degradation of microcapsules proceeds in three stages. The first starts at 140 °C near the melting point of PMMA due to leakage of the hardener from the microcapsules. The weight loss in the second and third stages, starting from 280 and 325 °C, corresponds to the stages of thermal decomposition of the shell.

K. Ning et al. [135] studied the effect of the size of microcapsules, small (33 ± 8 μm), medium (68 ± 21 μm), and large (198 ± 43 μm), at different contents (5, 10 and 15 wt.%). The healing liquid, consisting of a mixture of triethylene glycol dimethacrylate with N,N-dimethyl-*p*-toluidine, polymerizes upon initiation with benzoyl peroxide (0.5–2.0 wt.%). Fracture toughness (K_IC_) tests have shown that the highest self-healing efficiency (up to 76%) is achieved using 15% of the largest microcapsules. The efficiency of self-healing increases with increasing the concentration of the initiator, leading to a maximum value at 2.0 wt.%.

The system of “core-shell” microcapsules for the visual detection of mechanical damage is a conjugated cyclic olefin, 1,3,5,7-cyclooctatetraene, and a precursor of intensely colored polyacetylene. Effective encapsulation of the healing material requires a combination of poly(urea-formaldehyde) and polyurethane. Increasing the wall thickness of the outer shell and incorporating a core-side prepolymer improves the thermal stability and flowability of these capsules, which tend to leach and rupture as the shell wall thickness decreases. Capsules that burst in the presence of Grubbs’ ruthenium catalyst show an immediate color change from almost colorless to red-orange and deep purple over time, as well as a discoloration of thin films due to scratch damage [136].

T.E. Sadrabadi et al. [137] developed self-healing epoxy coatings containing two types of microcapsules, one with Epon 828 epoxy resin and the other with diethylenetriamine. The first type with a urea-formaldehyde shell was obtained by in situ polymerization; the second type was obtained by vacuum infiltration of an amine into nanoporous hollow glass microspheres (HGM). The process of obtaining the latter consisted of the chemical etching of HGM with dilute hydrofluoric acid. The optimal weight ratio of amine and epoxy microcapsules was 1:1, and the optimal healing efficiency was achieved at a total concentration of microcapsules of 15 wt.%.

### 4.2. Use of Hollow Glass Fibers

The method of self-healing composites using hollow glass fibers (HGF) can use a one-component system, when the healing agent is placed in a test tube, and the catalyst or hardener is contained in a matrix; or a two-component system, when both reagents are placed in different test tubes. During injury, some of these HGFs are degraded, initiating repair by healing, in which a healing agent enters the injury site and acts to mitigate the effects of matrix cracking and composite delamination and, most importantly, prevent further damage from spreading [32].

A typical technique for using HGF is demonstrated in the work of [138]. First, the self-healing agent SHA (unsaturated polyester resin) was mixed with 5% cobalt 2-ethylhexanoate and introduced into HGF while keeping the mixture in a desiccator. One end of these HGFs was immersed in SHA, and a suction force was applied to the other end; SHA penetrated the inside of HGF. A similar procedure was used to introduce the MEKP (2-butanone peroxide solution) hardener into HGF. HGFs were then removed and sealed at both ends with adhesive. HGFs filled with SHA and MEKP were glued together in a 2:1 ratio. A set of 20 bonded HGFs was placed on top of four layers of fiberglass exactly centered near the force application. Four layers of fiberglass were laid on top of these to obtain the required package thickness. The latter were cured by vacuum transfer molding the resin at room temperature for 24 h. The packages were then removed, and the specimens were cut out for testing.

**Figure 12:**
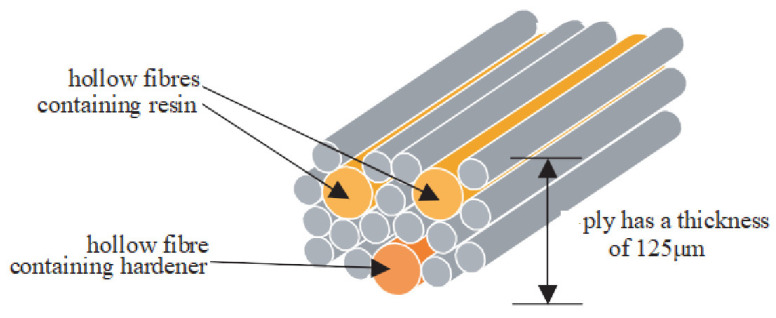
An arrangement of self-healing HGFs with resin and hardener placed within a layer of glass fibers in a stack sequence of a composite laminate. Fiberglass is shown in grey. Reproduced from [32] with permission from the Royal Society.

An example of HGF placement between laminate layers is shown in Figure 12.

The mechanism of healing is not fully understood, and some key questions regarding the extent and location of damage, the interaction of fractures with HGF, and the migration of healing agents remain unanswered.

**Figure 13:**
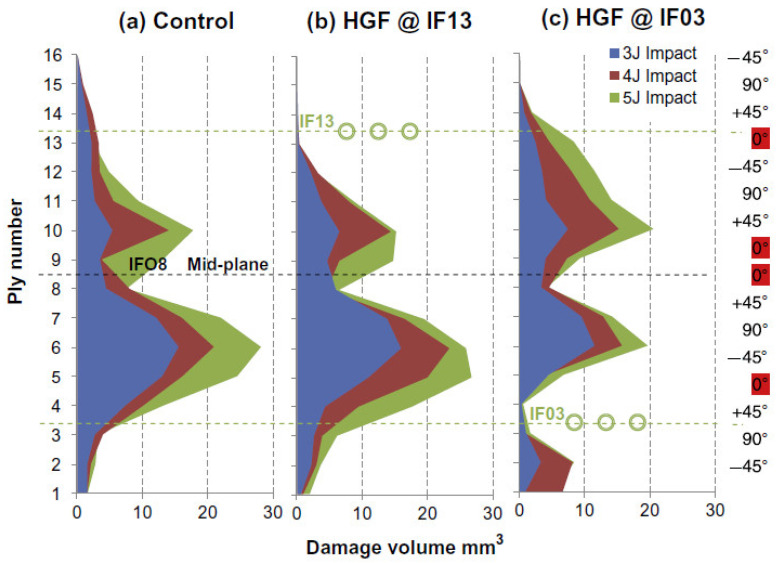
Mean damage volume per ply for (**a**) the control, (**b**) the HGF@IF13, and (**c**) the HGF@IF3 specimens impacted at 3, 4, and 5 J on ply 16. Reproduced from [139] with permission from Elsevier.

The authors of the work in [139] have shown that micro-X-ray computed tomography (μCT) can be used for the non-destructive evaluation of materials and structures and can thus answer some of these questions. In this study, X-ray μCT was used to quantify the volume and location of damage created in 16-ply quasi-isotropic carbon fiber reinforced plastic laminates by low-velocity impacts. Three different laminate configurations were considered: either laminates with unmodified carbon fiber-reinforced plastic or those containing an additional ply of HGF at the interface of the 3rd (HGF@IF3) or 13th (HGF@IF13) plies (Figure 13). The laminates were impacted with 3, 4 or 5 J using a drop weight from a tool tower.

As can be seen, the intensity of damage for different plies was different. The control samples (Figure 13a) contained two damage peaks, one in each half of the laminate, with the largest peak occurring in the lower half. The minima located in the center are associated with the practically absent interface between two plies (8 and 9) with a zero orientation. The choice of interface on which containers containing self-healing reagents are placed is critical. HGFs located between plies 13 and 14 had a minimal effect on damages (Figure 13c), whereas placement of HGFs between plies 3 and 4 had a significant healing effect (Figure 13b). Therefore, more HGFs can be destroyed if they are placed between more damaged plies.

### 4.3. Materials with Microvasculature

Unlike previous approaches, which were limited to repairing a single area of damage at a given location, systems containing a vasculature distribute the healing agent throughout the material, so that any part has access to the supply of the entire system. The concept of self-healing in a composite, based on the development of an extensive vascular network embedded in the laminate that can deliver a healing agent to damage sites, requires [140] that interfaces at which damage is most likely to be identified and then the network should be designed toward the self-healing of a particular material, and both dendritic [35] and lattice [141] channel architectures can be used.

In this case, one should keep in mind the location of the branches of the microvasculature relative to the reinforcing fibers and laminate layers. The case when the orientation of the vessels is perpendicular to the direction of the local fibers is the most unfavorable for the structural integrity of the composites [142], as this leads to the geometric distortion of the surrounding fibers and the formation of resin-rich pockets, which can be potential defects in the structural laminate. The technique of self-healing transverse vessels has been used in multilayer laminates to mitigate intralaminar damage by injecting low viscosity epoxy resins into the damage zone [37]. Under a static load, the rigidity was restored, and under a fatigue load, the mechanical properties turned out to be insufficient.

Most healing agents used for self-healing composite materials are two component systems. Therefore, it is possible to use two networks containing both components separately [141]. In another approach, solid catalyst particles (Grubbs) are included in the matrix, and the network is filled with a liquid healing agent (DCPD) [13,143].

The manufacture of a microvascular composite begins with the mechanized weaving of degradable fibers into three-dimensional woven blanks. The position, length, diameter, and curvature of the fibers varied depending on the design criteria [35]. In this case, the fibers used must be strong enough to withstand the process of mechanical weaving and vacuum impregnation. In addition, the fiber must remain solid during matrix cure (for example, up to 180 °C) but then be easily removed by depolymerization to a gaseous monomer at temperatures within a narrow range between the highest resin cure temperatures and the lowest thermal depolymerization temperatures (200–240 °C).

In the work of [144], vascular networks were introduced into the composite by evaporating poly(lactic acid) fibers by heating the sample above 200 °C in a vacuum. C.J. Norris et al. [145] used a wire with a low melting point (0.25- or 0.5-mm diameter, 60% Sn, 40% Pb alloy with a melting point of 188 °C) for this purpose. After the composite had cured, it was removed by heating at 190 °C in vacuum for 2 h.

In the works of [144,146], another method was proposed. When laying uncured prepreg bags, pieces of stainless-steel wire were inserted between the mid-length plies. The wire was located perpendicular to the orientation of the fibers and passed over the entire width of the samples. The prepreg was placed in vacuum bags and cured in an autoclave according to the cycle suggested by the manufacturer. Thereafter, the steel pieces were manually removed from the laminates, leaving the vessels with hollow channels in the laminates.

Advances in soft lithography have made it possible to create synthetic materials with microvascular networks [147]. Three-dimensional periodic scaffolds are made by applying ink in a layer-by-layer building sequence, followed by impregnation with epoxy resin. The resin is then cured, and the volatile ink is removed by heating the sample under vacuum to 70 °C.

**Figure 14:**
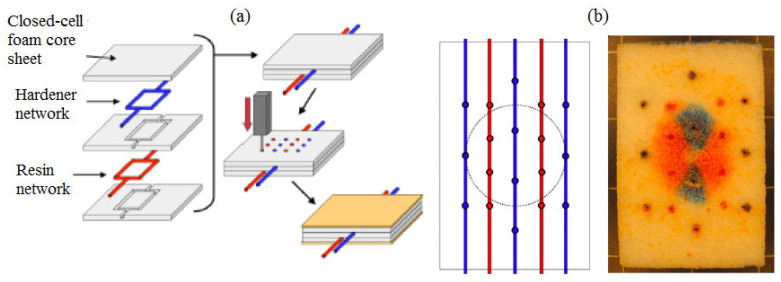
Illustration of self-healing after impact damage for the structure of fiber-reinforced polymers: (**a**) integration of the vasculature in composite sandwich panels and (**b**) damage healing (circle in the diagram) using a two-component adhesive delivered from an external reservoir. Reproduced from [148] with permission from IOP Publishing.

In all the above cases, the networks were filled with a healing agent in advance, prior to testing. C.J. Norris et al. [148] developed a trigger mechanism for filling a microvascular channel embedded in a multilayer composite material after damage to the latter (Figure 14). The trigger mechanism developed (Figure 14a) after impact damage (Figure 14b) triggered a DC motor-driven peristaltic pump through a solid-state relay using rudimentary software. The pump circulated the pre-mixed epoxy healing agent from an external reservoir (flow rate of 0.8 mL/min), delivering the healing agent to the damaged area within the specified time (5 min). With this approach to healing, an almost complete recovery of compressive strength after impact was achieved (average 94%).

The self-healing method using the vasculature has achieved impressive success, both in terms of identifying suitable healing agents and in terms of the preliminary design of the network, and it shows improved performance compared to other methods [38].

H. Tsilimigkra et al. [149] compared several self-healing methods. As potential self-healing systems, microcapsules with 5 or 10 wt.% vasculatures made using wax and steel wire and 7 wt.% additives of thermoplastic polymers were introduced into the composite. Table 1 shows the values of the self-healing efficiency modulo and the three-point bending strength for each of the systems.

As can be seen, the restoration of properties was not fully implemented, and the degree of its implementation depends on the method used. The use of microcapsules is limited by the fact that after the rupture of the capsule, only one healing cycle is possible. The implementation of the vasculature leads to the formation of voids, which themselves can be considered damage. When using thermoplastics, an external mechanism is required to start the healing process, which is difficult to implement in the case of complexly organized composites.

## 5. Self-Healing Metallopolymers

Macromolecules containing transition metals are promising candidates for the development of new materials due to the metal center, which imparts several functional properties to the polymer, such as being optical, catalytic, magnetic, self-healing, etc. [150,151,152,153,154,155,156,157,158], due to which metallopolymers are widely used as polymeric LEDs, catalysts, magnetic, luminescent and sensor materials, drug delivery systems, and in other biomedical applications [159,160,161,162].

**Figure 15:**
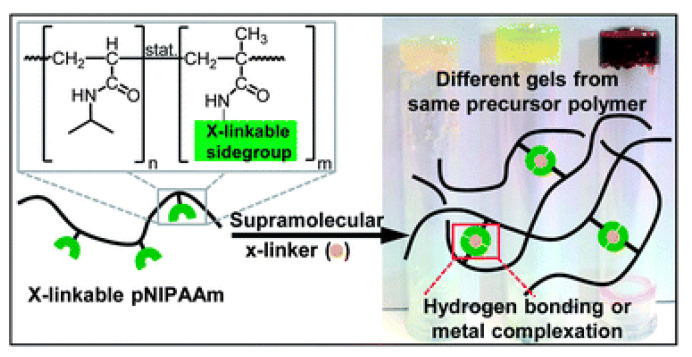
Scheme of the formation of non-covalent bonds in supramolecular systems. Reproduced from [170] with permission from the Royal Society of Chemistry.

The self-healing properties of metallopolymers have been intensively studied in recent years [163,164,165,166]. Among the self-healing polymers developed to date, a significant place is occupied by metallosupramolecular polymers formed as a result of M–L complexation. Due to the reversibility, high stability, and rate of formation of coordination complexes, this strategy can be used to synthesize polymer structures capable of stimulated self-assembly, self-repair, and mechanical rearrangement using redox processes, as well as changes in pH or temperature, etc. [167,168,169]. The supramolecular assembly is a macromolecular network due to non-covalent bonds, such as hydrogen bonds, complexation with transition metals, hydrophobic interactions, or π–π stacking (Figure 15) [170].

These interactions are strong enough to form physical bonds that break easily and reversibly under adverse conditions, making them sensitive to stimuli and useful in applications requiring, for example, self-healing or shape memory. In principle, two different molecular mechanisms may contribute to the overall healing of such polymer systems. On the one hand, ionic clusters, the formation of which is typical of ionomeric polymers, can function as a reversible element providing crack closure [171,172,173]. This process is facilitated by the often positively charged metal complexes present in the more hydrophobic polymer, which can also lead to phase separation. On the other hand, the dynamic M–L interaction can be stimulated, for example, by light or heat, which leads to the opening of the supramolecular bond and, therefore, to mobility, which is a necessary condition for healing. In principle, both interactions may play a role in the healing process.

### 5.1. With Terpyridine Ligands

**Figure 16:**
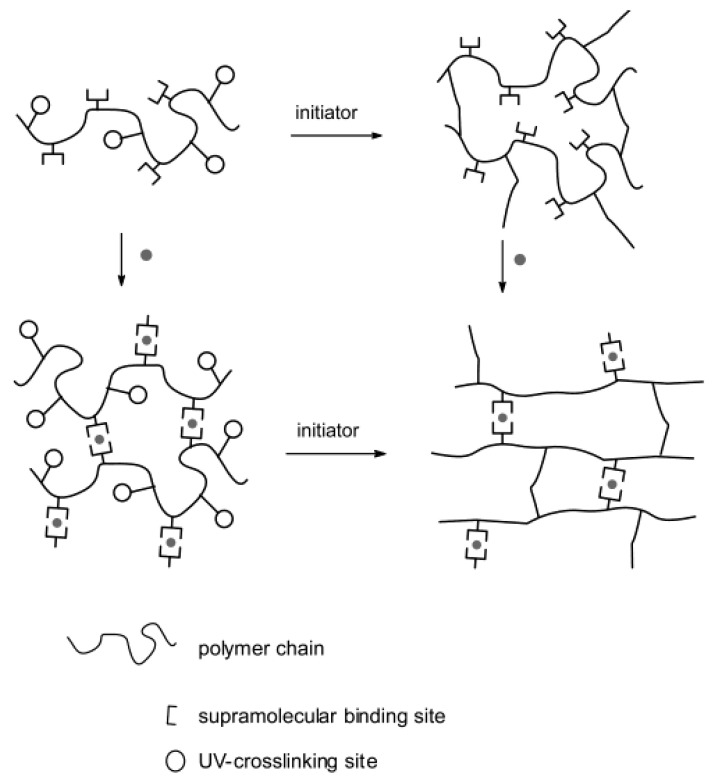
Scheme of two-stage formation of covalent and supramolecular crosslinks during terpolymer synthesis. Reprinted with permission from [178]. Copyright (2003) American Chemical Society.

Most of the M–L complexes used to create self-healing materials include terpyridine-metal complexes [168,174,175,176]. In particular, a crosslinked metallopolymer system based on the chemistry of the iron(II)-bis-terpyridine (M–L) complex was demonstrated [177]. Various polymers have been synthesized from terpyridine methacrylate (6-(2,2′:6′,2″-terpyridin-4′-yloxy)hexyl methacrylate) monomers copolymerized with three different methacrylate monomers (methyl methacrylate, butyl methacrylate, and lauryl methacrylate) by living polymerization This approach makes it possible to control the proportion of crosslinkable units and, as a result, the thermal and mechanical behavior of polymer networks. The best self-healing results were observed at 100 °C for an organogel based on a lauryl methacrylate copolymer, whereas a metallopolymer gel based on methyl methacrylate did not fully self-heal due to higher glass transition temperatures and, accordingly, less flexibility of comonomer units. When heated, the degree of crosslinking is constant, which indicates that the self-healing mechanism is not based on the destruction of the terpyridine complex. The authors attribute a more probable self-healing mechanism to the considered metallopolymers with the formation of ionic clusters. An interesting approach was proposed in the work of [178] for the synthesis of an acrylate terpolymer containing a terpyridine group as a supramolecular fragment and an oxetane fragment capable of covalently crosslinking (Figure 16). The obtained supramolecular polymers, in which non-covalent interactions are combined with covalently crosslinked fragments, open great opportunities for the development of new materials with self-healing properties.

The thermally induced mechanism of self-healing of metallosupramolecular polymers based on bisterpyridine complexes of iron(II) sulfate and cadmium(II) sulfate was used. It turned out that self-healing mechanisms based on the partial destruction of crosslinking complexes are decisive. Promising photocatalytic systems for solar energy conversion are polymers based on terpyridine and their ruthenium complexes, in which M–L interactions can be reversibly destroyed and restored [179]. The initially active photocatalyst system becomes inactive after the destruction of the complex, but its activity is restored again by self-assembly, and this process can be repeated several times.

**Figure 17:**
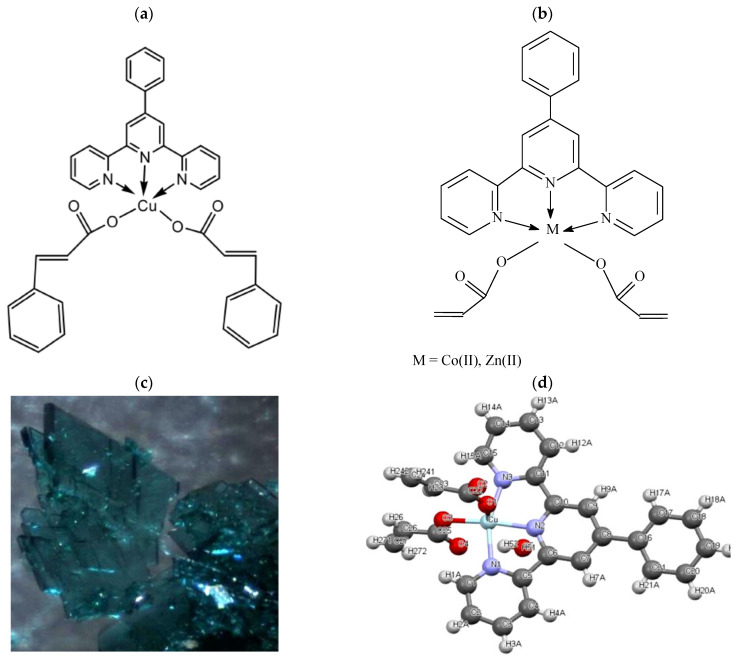
Schematic representation of synthesized MCMs with pendent 4-phenyl-2,2’:6’,2″-terpyridine ligand (**a**)—CuCinnamatePhTpy, (**b**)—ZnAcrylatePhTpy and CoAcrylatePhTpy, (**c**)—appearance of crystals of the CuAcrylatePhTpy complex in the form of triclinic pyramids (prisms) and its molecular structure (**d**). Reprinted from [187] with permission from Elsevier.

In recent years, a direction based on the preparation of metal-containing monomers (MCMs) with terpyridine fragments and metallocopolymers based on them, which have self-healing properties, has been intensively developed [180,181,182]. A series of mixed-ligand complexes based on unsaturated carboxylic acids (maleic, acrylic, β-phenylacrylic, and itaconic), as well as cobalt(II) [183], nickel(II) [184,185], copper(II), and zinc(II) [186,187] ions were obtained by the interaction of metal-containing monomers with 4-phenyl-2,2′:6′,2″-terpyridine (Figure 17). MCMs of this type are of interest from the point of view of the presence of M–L coordination bonds and the stereochemistry of the chelate site, as well as because of the effect of the metal ion on the reactivity of the double bond during polymerization and the possibility of creating a wide range of polymer architectures, including metallosupramolecular polymers with self-healing properties.

**Figure 18:**
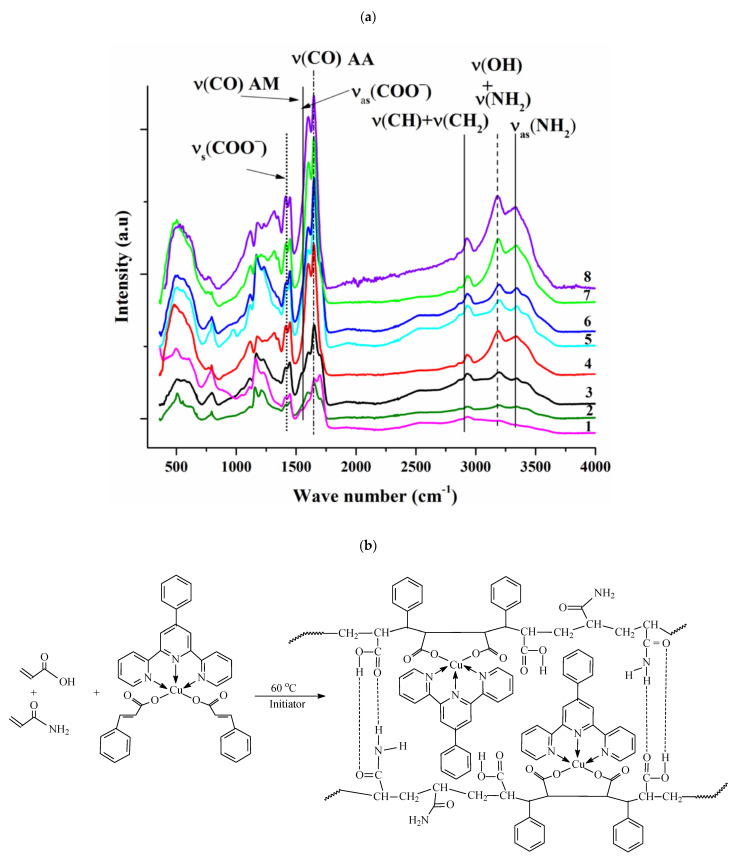

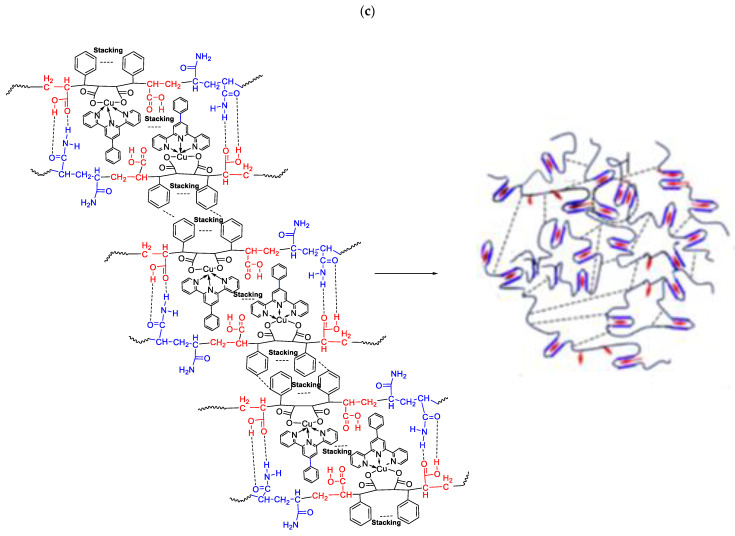
(**a**) IR spectra of copolymers isolated from gel solutions by precipitation in acetone and dried in air: 1—[AM/AA/CuCinnamatePhTpy = 14.5/84.5/1]; 2—[AM/AA = 50/50] 50% sol.; 3—[AM/AA/ZnAcrylatePhTpy = 49.5/49.5/1] 50% sol.; 4—[AM/AA/ CuCinnamatePhTpy = 84.5/14.5/1]; 5—[AM/AA/CuCinnamatePhTpy = 49.5/49.5/1] 50% sol.; 6—[AM/AA/CuCinnamatePhTpy = 49.5/49.5/1]; 7—[AM/AA/ZnAcrylatePhTpy = 84.5/14.5/1]; 8—[AM/AA/CuCinnamatePhTpy = 84.5/14.25/1.25]; (**b**) a scheme of a radical polymerization of AA, AM, CuAcrylatePhTpy; (**c**) schematic representation of the structure of the obtained copolymer P(AM-co-AA-co- MCMPhTpy). Data of [180].

Ternary copolymers based on MCMs with phenylterpyridine ligands (MCMPhTpy), successfully obtained by radical copolymerization of MCMPhTpy with acrylic acid (AA) and acrylamide (AM), according to IR spectra, exhibit a developed network of hydrogen, coordination, and π–π bonds that play a decisive role in the manifestation of the self-healing properties of the obtained metallocopolymers (Figure 18) [180].

**Figure 19:**
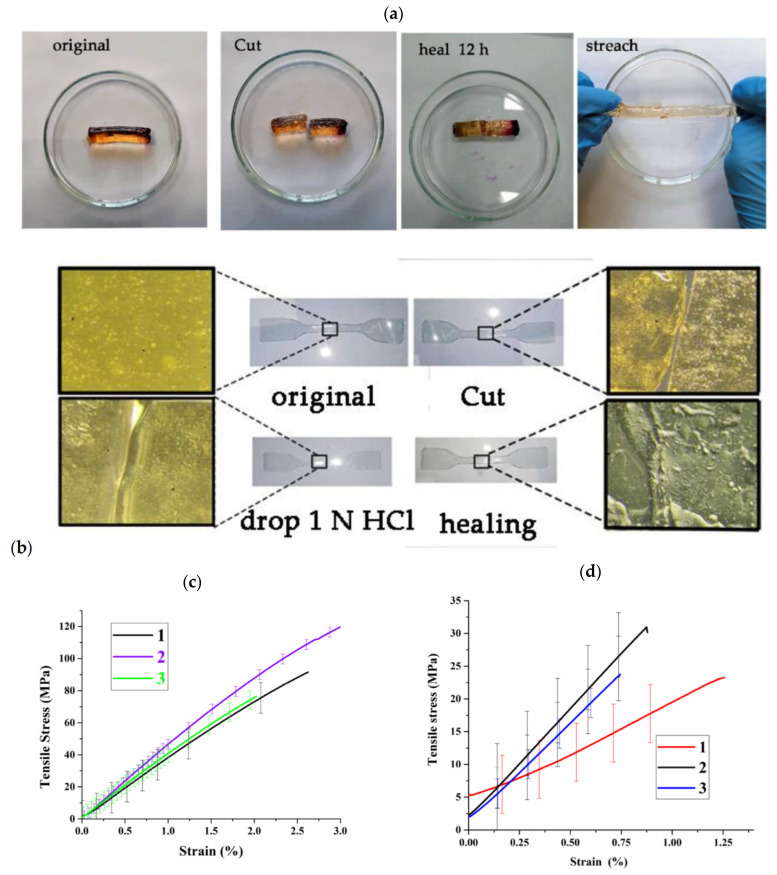
(**a**) Photographs of the healing process of the gel 13 ((AM/AA/ZnAcrylatePhTpy = 49.5/49.5/1) in 50 wt.% sol), demonstrating its mechanical deformation and recoverability. (**b**) Optical microscope photographs of the healing process of dogbone shape samples of copolymer 8–(AM/AA/CuCinnamatePhTpy = 84.5/14.5/1])using an acidic solution (1 drop of 0.1 M HCl solution). (**c**) Effect of MCM addition on physical and mechanical properties of P(AM-co-AA): 1—[AM/AA = 50/50]; 2—(AM/AA/ZnAcrylatePhTpy = 49.5/49.5/1); 3—(AM/AA/CuCinnamatePhTpy = 49.5/49.5/1) (AA/AM (mol) = 1) prepared in 80 wt.% sol. (**d**) Mechanical properties of copolymers after healing for 12 h: 1—(AM/AA/CuCinnamatePhTpy = 14.5/84.5/1); 2—(AM/AA/CuCinnamatePhTpy = 84.5/14.5/1); 3—(AM/AA/CuCinnamatePhTpy = 49.5/49.5/1). Data of [180].

Along with the fact that metallocopolymer films have good mechanical properties, they also show self-healing properties at room temperature with the addition of one drop of 0.1 M HCl solution, as shown in Figure 19 [177]. The efficiency of the self-healing of mechanical properties depends on the composition of the copolymer and can reach 78–80% (Figure 19). It has been shown that in copolymers with a high content of acrylamide, the healing efficiency is higher. This behavior seems to be due to the fact that with an increase in the acrylamide content in the metallocopolymer, their mechanical properties weaken, which was also reported in the work of [188]. The system becomes more dynamic, which in turn leads to improved self-healing properties, as many studies have shown [189].

Thus, the search for autonomous self-healing is still a challenge for materials that exhibit both improved mechanical properties and high self-healing abilities.

### 5.2. Llocopolymer Hydrogels

The phenomenon of self-healing hydrogels is due to the dynamic nature of the bonds connecting polymer structures to each other with the formation of a crosslinked hydrophilic system. In biological systems, which are inherent in self-healing, ionic, hydrogen, and M–L coordination bonds are extremely common. The use of several types of interactions of functional groups in polymer chains and various metal cations during the synthesis of the material makes it possible to control the strength of coordination bonds and, accordingly, the adhesion, strength, and elasticity of the resulting gel.

For example, amide and carboxyl groups form a system of hydrogen bonds in the resulting hydrogel; in addition, they allow additional crosslinking of the hydrogel structure by introducing a complexing metal cation [190,191]. In several works, when creating hydrogels, the addition of a water-soluble polymer to the initial monomer mixture was used, which creates additional crosslinks in the resulting hydrogel due to hydrogen bonds between polymer chains [192,193]. In recent years, much attention has been paid to the creation of self-healing hydrogels based on natural polysaccharides, such as alginic acid, xanthan, and chitosan, due to their stability, biocompatibility, and biodegradability. Of particular interest is chitosan obtained by the deacetylation of chitin, the second most abundant polysaccharide in nature after cellulose. Its antibacterial activity and mechanical properties make it possible to consider chitosan as a basis for creating materials for a wide range of applications [194]. The self-healing mechanism of hydrogels based on chitosan can be based on the reversibility of the formation of Schiff bases [195] or on non-covalent interactions [196,197]. Examples of two-component systems are known, such as chitosan/PVA, chitosan/alginate, and chitosan/polyacrylic acid [198,199,200]. When dissolved in water, due to hydrogen bonds, hydrophobic, electrostatic, and other types of non-covalent interactions, a three-dimensional network is formed, in which chitosan plays the role of a skeleton. There are examples of introducing graphene oxide NPs or Fe^3+^ ions into such networks, including the production of self-healing hydrogels [201,202].

It is known that traditional synthetic polymer hydrogels usually have low mechanical strength, minimal impact strength, and low healing ability [203]. This is largely due to their single network structure, which leads to internal structural heterogeneity due to the irregular distribution of crosslink sites and the lack of effective energy dissipation mechanisms. In addition, they are difficult to process. In recent years, significant progress has been made in attempts to obtain polymer hydrogels with improved mechanical characteristics, such as double-network gels, interpenetrating networks, macromolecular microspheres, nanocomposite hydrogels, crosslinked gels (dual crosslink gels), etc. [204,205]. Therefore, an important direction in the development of new hydrogels is the inclusion of non-covalent bonds as crosslinks in gel polymer networks to dissipate energy under loads and improve the physical and mechanical characteristics of the final material [206,207]. Due to the nature of ionic and hydrogen bonds, hydrogels based on them are more amenable to processing and are potentially capable of self-healing. One type of non-covalent interaction is M–L coordination bonds, which are extremely common in biological systems. The use of various metal cations makes it possible to control the strength of coordination bonds and, accordingly, the adhesion, strength, and elasticity of the resulting material. In such supramolecular systems, covalent bonds serve as the basis for creating the primary structure responsible for the integrity of hydrogels, and non-covalent bonds (for example, hydrogen bonds, coordination bonds, and hydrophobic interactions) act as sacrificial bonds that can be reversibly broken and reformed. As a result such processes, the mechanical characteristics of damaged systems can be restored. Metallopolymer gels were obtained by supramolecular assembly by reacting Ni^2+^, Co^2+^, Fe^2+^, and Zn^2+^ ions with a terpyridine-functionalized norbornene polymer [208].

Several strategies have been successfully applied in the rational design of metallosupramolecular polymer networks and gels [209,210], which can be divided into two categories depending on how crosslinks are formed within the supramolecular network. The first criterion includes a combination of ditopic ligands and metal ions that can bind more than two ligands, resulting in tris- or higher complexes. The metal ions function as branching points or as crosslinking agents in the polymer network. Based on transition metal ions and a ditopic oligomer, a supramolecular gel with a stimulus-sensitive response to temperature, chemical, and mechanical external influences was obtained [211]. The second method involves the combination of multitopic ligands and metal ions to form bis-chelate complexes. A typical example of such a gel is given in the works of [212,213]. The coordination motifs are based on poly(4-vinylpyridine) and a bifunctional palladium(II) complex.

## 6. Summary and Future Outlook

Self-healing polymers and polymer composites are of growing interest to researchers. The successful development of self-healing polymeric materials opens great opportunities for expanding their application in the production of structural and critical composites. It is clear that both molecular and structural approaches are applicable to the self-healing of thermoplastic and thermosetting polymers, as well as to the repair of composites and structures.

**Figure 20:**
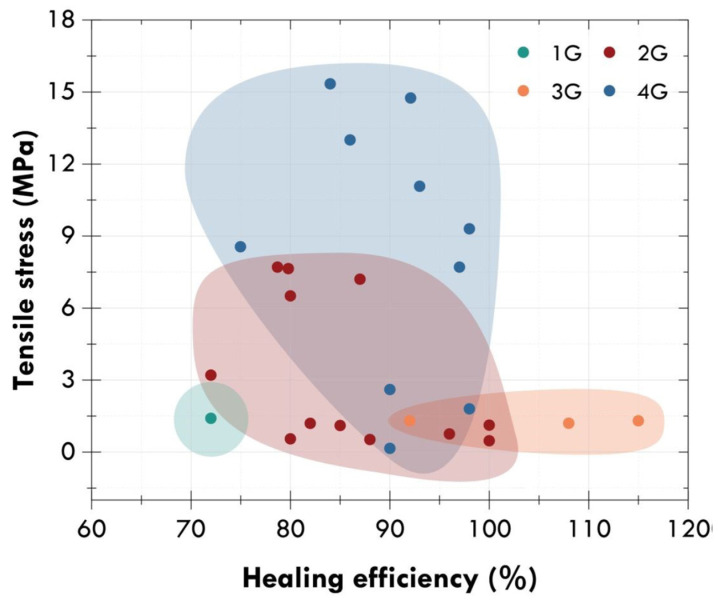
Diagram of four generations (G) of self-healing elastomers. Reproduced from Ref. [215] with permission from the Royal Society of Chemistry.

According to S. Van der Zwaag [214], the generational classification of self-healing polymers based on the healing mechanism correlates with historical development. The first generation used encapsulation of external healing agents; the second, based on intrinsic mechanisms, applied reversible chemistry; in the third generation, the healing agent is embedded in the vasculature. Recently, the fourth generation of self-healing materials has been developed, combining covalent bonds and non-covalent interactions, which provides an optimal balance between mechanical characteristics and repairability (Figure 20) [215].

Intrinsic methods use the phenomenon of reversible polymerization, which is triggered by external factors such as heat, light, etc. HGF- and capsule-based mechanisms are suitable for healing small fractures, and vasculature can also be used for large damages. The integration of sensors into the self-healing mechanism can increase the healing potential (see, for example, [66]).

Recently, advances have been made in the production of self-healing materials suitable for structural and other commercial applications [216]. For example, in the work of [217], the self-healing of high-impact polystyrene based on microcapsules for 3D printing and increasing the service life of durable composites was studied. A material with 3D printed vasculature has excellent self-healing potential [218]. The use of NPs to increase the efficiency of self-healing is becoming more widespread [58,91,97,219,220].

Materials that are capable of autonomously detecting and repairing damage at the initial level have great potential and application, especially in cases in which it is necessary to ensure the long-term reliability of materials in hard-to-reach places.

The main disadvantage of the process with an external initiation of self-healing due to the introduction of restoring components of the capsule is the possibility of carrying out only a single “regeneration”.

The development of technologies for capsule systems to eliminate the problem of a single “healing” is aimed at embedding hollow fibers (capillaries) with liquid fillers into the matrix material instead of capsules. Their advantage also lies in the possibility of various 2D and 3D weaving of capillaries to increase the ability of the composite to self-heal. The segregated microvascular channels can provide sufficient volumes of a healing agent to completely infuse a significant impact injury. Self-healing systems with hollow fibers also do not completely solve the problem of obtaining a reusable “self-healing” effect, since the components that ensure the healing of such a composite material are consumed and do not occur repeatedly in the required amount. Therefore, the further development of this technology is associated with the supply of the necessary components or their pumping (in the case of a two-component liquid scheme), which is a direct analogy with the self-healing of biological tissues. This pattern of healing is called the “vascular system”. Recovery efficiency can be achieved, as discussed above, using elongated capsules.

Supramolecular polymers offer key advantages over alternative approaches, as these materials can typically withstand multiple healing cycles without a significant loss in performance due to fully reversible non-covalent interactions. In recent years, sensitive supramolecular materials using metal–ligand interactions have been widely studied. Especially promising are systems that combine other non-covalent interactions, such as hydrogen bonds or stacking interactions. In the damaged state, the viscosity of metal-supramolecular polymers is significantly reduced, which allows the material to flow and form new supramolecular bonds, resulting in the rapid and efficient healing of the material.

At the same time, these versatile structural elements can also provide an upper limit to the strength and application of these materials, i.e., some balance is needed between the dynamic nature of the reversible bonds and the mechanical or thermal stability of the materials. It can be assumed that an increase in the strength of supramolecular interactions will improve the mechanical properties, but it will be necessary to use more stringent healing conditions, for example, higher temperatures, etc.

In self-healing metallopolymers, there are two reversible interactions: non-directional ionic interactions between positively charged metallocomplexes and negatively charged counterions and directed M–L interactions. For example, thermo-, photo-, and chemo-sensitive shape memory has been achieved using reversible M–L bonds [221]. If weak and dynamic metallocomplexes are used, then the destruction and rearrangement of the complexes can provide the necessary mobility. In addition, ionic metallocomplexes can cause the formation of ionic clusters, which also contribute to the mobility of the system.

Self-healing materials can increase the lifespan, safety, and energy efficiency of synthetic materials and help reduce the environmental impact of synthetic materials, and their applications can range from engineering materials and polymer coatings to biomedical applications [222,223,224].

Close cooperation between chemists, materials scientists, and computer scientists requires the effective application of all these approaches to self-healing on an industrial scale.

## Figures and Tables

**Table 1:** Self-healing efficiency, %.

System	Module	Strength
Microcapsules 5 wt.%	72	88
Microcapsules 10 wt.%	57	87
Vasculatures (wax)	70	70
Vasculatures (wire)	74	76
Polyethylene terephthalate	52	49
Nylon doped with 10% multiwalled carbon nanotubes	38	36

## Data Availability

Not applicable.
